# Excellent Persistent Near‐Infrared Room Temperature Phosphorescence from Highly Efficient Host–Guest Systems

**DOI:** 10.1002/advs.202402846

**Published:** 2024-05-17

**Authors:** Shuhui Li, Juqing Gu, Jiaqiang Wang, Wentao Yuan, Guigui Ye, Likai Yuan, Qiuyan Liao, Le Wang, Zhen Li, Qianqian Li

**Affiliations:** ^1^ Hubei Key Lab on Organic and Polymeric Opto‐Electronic Materials Department of Chemistry Wuhan University Wuhan 430072 China; ^2^ TaiKang Center for Life and Medical Sciences Wuhan University Wuhan 430072 China

**Keywords:** energy transfer, intramolecular charge transfer, near‐infrared phosphorescence, prolonged lifetime, room temperature phosphorescence

## Abstract

Organic near‐infrared (NIR) room temperature phosphorescence (RTP) materials become a hot topic in bioimaging and biosensing for the large penetration depth and high signal‐to‐background ratio (SBR). However, it is challenging to achieve persistent NIR phosphorescence for severe nonradiative transitions by energy‐gap law. Herein, a universal system with persistent NIR RTP is built by visible (host) and NIR phosphorescence (guest) materials, which can efficiently suppress the nonradiative transitions by rigid environment of crystalline host materials with good matching, and further promote phosphorescence emission by the additional phosphorescence resonance energy transfer (≈100%) between them. The persistent NIR phosphorescence with ten‐folds enhancement of RTP lifetimes, compared to those of guest luminogens, can be achieved by modulation of aggregated structures of host–guest systems. This work provides a convenient way to largely prolong the phosphorescence lifetimes of various NIR luminogens, promoting their application in afterglow imaging with deeper penetration and higher SBRs.

## Graphical abstract

The persistent near‐infrared (NIR) room temperature phosphorescence (RTP) is achieved by doping NIR phosphors (guest) into visible RTP materials (host) as a new type of host–guest system, with ten‐folds increase of RTP lifetimes. The spatial matching of hosts and guests is proven as the crucial factor for the first time, which can efficiently suppress nonradiative transitions by space restriction, promoting RTP emission.

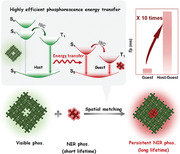

## 1 Introduction

**Figure 1:**
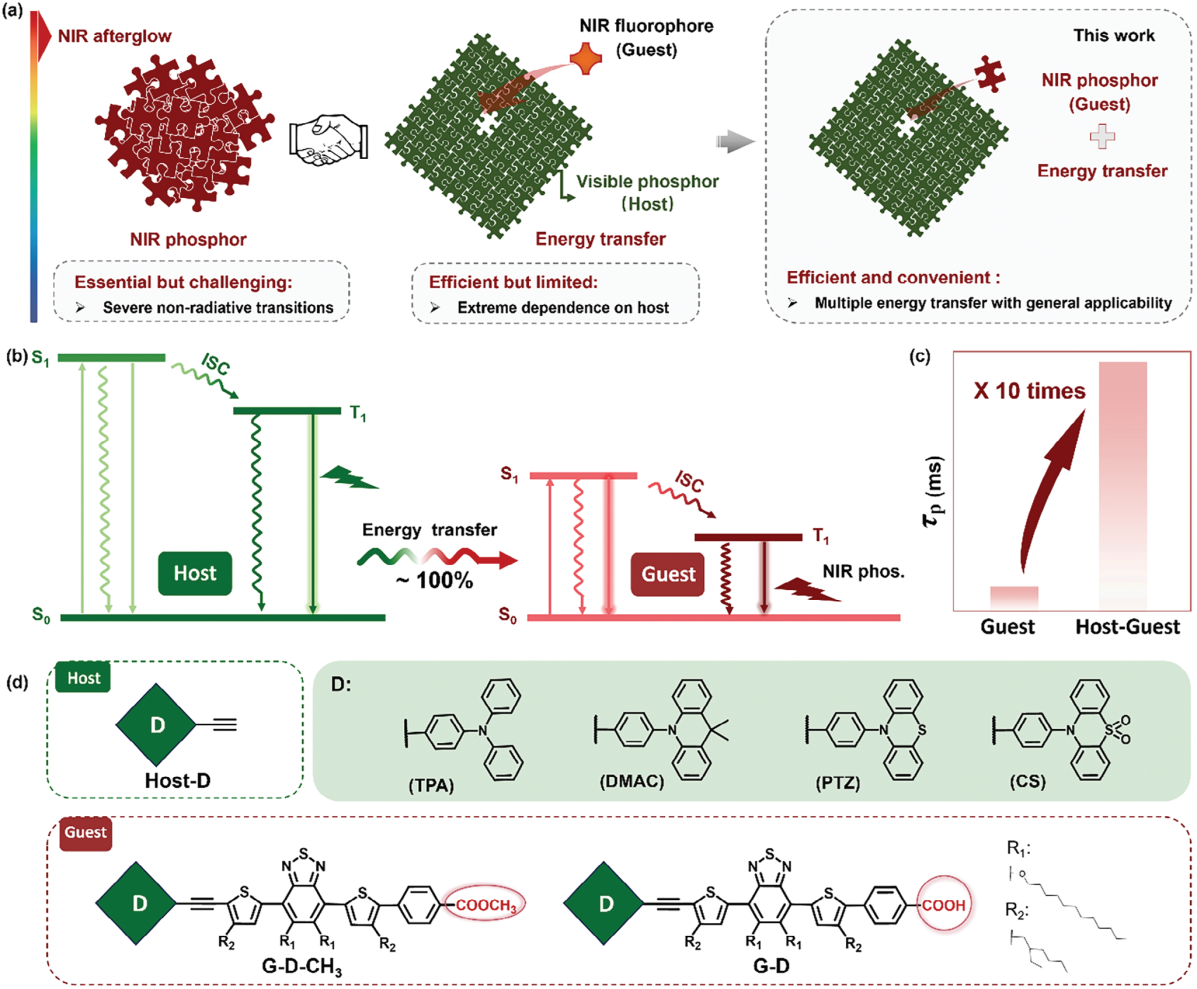
a) Strategies to construct the persistent NIR phosphorescence of organic materials at room temperature. Previous work: organic NIR phosphors as the single component, and the phosphorescence resonance energy transfer between visible RTP materials and NIR fluorophores. This work: the phosphorescence resonance energy transfer between visible and NIR RTP materials. b) Jablonski diagram of host‐guest systems by organic visible and NIR RTP materials. c) The enhancement of RTP lifetimes from NIR phosphors as guests to host–guest systems by visible and NIR RTP materials. d) Molecular structures of hosts and guest materials with various electron donors and ending groups.

Organic room temperature phosphorescence (RTP) materials have received wide attention and made great progress in bio‐applications for good biocompatibility and high signal‐to‐background ratio (SBR) by nearly no background interference.^[^ ^1^ ^]^ With the aim to further improve the spatiotemporal resolution, penetration depth, and sensitivity of afterglow imaging, the extended wavelength of RTP emission to near‐infrared (NIR) optical window is urgently needed.^[^ ^2^ ^]^ Until now, the NIR RTP emission is mainly generated by metal complexes, including Ir(III) and Pt(II), which suffer from complicated syntheses, high cost, and biotoxicity.^[^ ^3^ ^]^ Recently, organic luminogens with strong intramolecular charge transfer (ICT) and/or special packing modes have been explored to extend the phosphorescence wavelength to the NIR region.^[^ ^4^ ^]^ However, the RTP lifetimes of these NIR luminogens are usually in the region of 0.03–3.69 ms (**Figure** **1**a), due to the lack of molecular design guidance. The short phosphorescence lifetimes are mainly related to the sensitive triplet excited states with low energy levels, which can be easily quenched by the nonradiative transitions, the unfavorable aggregated structures, and the possible collision by the oxygen and moisture in the environment.^[^ ^5^ ^]^ Thanks to the pioneer works of Gabellieri^[^ ^6a^ ^]^ and George,^[^ ^6b^ ^]^ phosphorescence energy transfer from RTP host to fluorescence guest could realize NIR afterglow. Accordingly, we have prepared a host–guest NIR afterglow system, which has been successfully applied in in vivo bioimaging of living mice for the first time. Later, some other excellent works have been reported. In general, benzophenone (BP) and triphenylamine (TPA) derivatives with green RTP emission have been employed as host materials, while porphyrin derivatives with NIR fluorescence act as the guest ones (Figure 1a).^[^ ^7^ ^]^ However, as the long lifetimes of NIR afterglow are totally from the triplet excited states of host materials, not the guest ones with NIR emission, the duration of NIR afterglow is heavily dependent on the initial RTP lifetimes of hosts and the efficiency of energy transfer. So far, limited examples with completely matched photophysical properties have been reported.^[^ ^8^ ^]^

Prompted by the above results and their still existing disadvantages, the energy transfer from RTP host to RTP guest deserves to be explored, to achieve better performance. As shown in the right picture of Figure 1a, the embedding of NIR phosphors into the host matrix can suppress the possible quenching effect by their unfavorable aggregation behaviors of themselves, and inhibit the non‐radiative transitions by rigid crystalline state of host molecules, together with the possible intermolecular interactions and π‐coupling between host and guest materials. Also, the afterglow emission is directly from the triplet excited states of RTP guests, assuring the much longer lifetimes independent of those of hosts. Thus, NIR afterglow can be promoted and prolonged by both stabilizations of triplet excited states and multiple energy transfer processes (Figure 1b), including singlet‐to‐singlet, triplet‐to‐triplet, and triplet‐to‐singlet ones.^[^ ^9^ ^]^

Considered with these, herein, a series of organic NIR phosphors with benzothiadiazole as the electronic acceptor (A), and various RTP building blocks, including TPA, 9,9‐dimethyl‐10‐phenyl‐9,10‐dihydroacridine (DMAC), 10‐phenyl‐10H‐phenothiazine (PTZ) and 10‐phenyl‐10H‐phenothiazine 5,5‐dioxide (CS) as the electron donors (D) have been designed and synthesized to strengthen the ICT effect. Also, carboxylic acid (‐COOH) has been incorporated as the anchoring group to increase the intermolecular interactions by the formation of hydrogen bonding (luminogen G‐D), while methyl ester (−COOCH_3_) without active hydrogen was added as the ending group for comparison (luminogen G‐D‐CH_3_) (Figure 1d). All of them demonstrated obvious NIR phosphorescence with the maximum emission wavelengths extended to 750 nm and onset wavelengths to 800 nm. Furthermore, with the employment of these D moieties with green RTP emission as the host materials (Host‐D), and these NIR phosphors with D‐A structures as guest materials, the resultant host–guest systems exhibited much improved NIR phosphorescence (Figure 1c). Excitedly, thanks to the highly efficient energy transfer (≈100%) and the suppression of non‐radiative transitions by the space restriction effect and strong intermolecular interactions, the corresponding RTP lifetimes can increase to 492 ms, ten‐folds enhancement than those of guest one. More importantly, various systems consisting by visible and NIR phosphorescence materials demonstrated bright NIR phosphorescence with prolonged lifetimes, indicating the universality of this kind of host–guest system, affording an efficient strategy to achieve the persistent NIR phosphorescence with the in vivo afterglow imaging of deeper issues in living mice possessing high SBR. Furthermore, the detailed analysis of the host and guest aggregate structures, well explained the inherent mechanism with the 1+1≻2, offering the guiding rule for the selection of matched RTP host and RTP guest for higher performance NIR afterglow.

## 2 Results and Discussion

The conjugated systems of NIR luminogens with strong electronic pull‐push properties were synthesized by Sonogashira coupling between alkynyl moieties substituted to TPA, DMAC, PTZ, and CS as D moieties, and thiophene‐benzothiazole‐thiophen (BT) skeletons as π‐A‐π type with the bromine atom serving as the reactive site. The ending groups of ─COOCH_3_ and ─COOH were incorporated by Suzuki coupling, resulting in the eight targets NIR luminogens of G‐D‐CH_3_ and G─D as the guest materials, respectively. Also, multiple alkyl/alkoxy chains with linear and branched structures have been linked to the side of conjugated skeletons, with the aim to adjust the aggregation behaviors of these luminogens, form multiple C─H…π and C─H…X (X represents heteroatoms, such as N, O and S) interactions among alkyl chains and/or conjugated skeletons, and adjust of molecular conformations by intramolecular O…S interactions. Also, they can form a hydrophobic environment in aqueous solutions, which can inhibit the possible quenching effect by water and oxygen, beneficial to in vivo phosphorescence imaging. Accordingly, the donor moieties were employed as the host materials (Host‐D) with the addition of alkynyl as the ending group to strengthen the intermolecular interactions. These target luminogens of Host‐D, G‐D‐CH_3_, and G‐D have been well characterized by ^1^H NMR, ^13^C NMR, and MS (EI or MALDI‐TOF).

**Figure 2:**
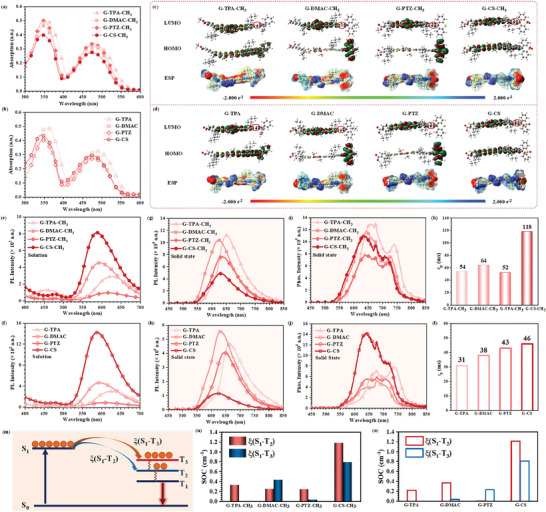
a) UV–vis absorption spectra of G‐D‐CH_3_ series in THF solution with the concentration of 1 × 10^−5^ mol L^−1^. b) UV–vis absorption spectra of G‐D series in THF solution with the concentration of 1 × 10^−5^ mol L^−1^. c) LUMOs, HOMOs, and ESP surfaces of G‐D‐CH_3_ series as isolated state. d) LUMOs, HOMOs, and ESP surfaces of G‐D series at isolated state. e) PL spectra of G‐D‐CH_3_ series in THF solution with the concentration of 1 × 10^−5^ mol L^−1^. f) PL spectra of G‐D series in THF solution with the concentration of 1 × 10^−5^ mol L^−1^. g) PL spectra of G‐D‐CH_3_ series at solid states at room temperature. h) PL spectra of G‐D series at solid states at room temperature. i) Phosphorescence spectra of G‐D‐CH_3_ series at solid states at room temperature. j) Phosphorescence spectra of G‐D series at solid states at room temperature. k) Phosphorescence lifetimes of G‐D‐CH_3_ at solid states at room temperature. l) Phosphorescence lifetimes of G‐D series at solid states at room temperature. m) Scheme diagram of ISC process from lowest singlet excited state (S_1_) to triplet excited states (T_n_), to generate phosphorescence emission. n) The SOC constants of G‐D‐CH_3_ series for S_1_→T_2_ and S_1_→T_3_ processes. o) The SOC constants of G‐D series for S_1_→T_2_ and S_1_→T_3_ processes.

For luminogens G‐D‐CH_3_ and G‐D, the broad absorption spectra ranging from 300 to 600 nm can be obtained for their strong ICT effect (**Figure** **2**a,b). Among them, the maximum absorption wavelengths of G‐TPA‐CH_3_ and G‐TPA were the largest ones at 487 and 485 nm, respectively, indicating the strong electron‐donating ability of TPA as D moiety, while the different ending groups have an ignorable electronic effect on the conjugated systems. Since all of these D moieties contained the same nitrogen (N) atom as the electron‐rich center, their different electron‐donating abilities were mainly related to the molecular configurations, especially the conjugation degree of the involved phenyl (red circles in Figure 2c,d) in conjugated systems and N atom, as well as the additional electronic effect by other heteroatoms. This case can be partially illustrated by Density functional theory (DFT) calculation with the Gaussian 09 program.^[^ ^10^ ^]^ As shown in Figure 2c, for DMAC, PTZ and CS moieties, the perpendicular configuration of 9,9‐dimethylacridan, phenothiazine, phenothiazine 5,5‐dioxide and involved phenyl can be observed, indicating the poor conjugated effect, while the conjugated effect can be increased in TPA moiety for the smaller dihedral angles of 66.83° and 67.08° for the involved phenyl and other phenyl units in TPA, respectively (Figure S1, Supporting Information). It can be further confirmed by their electrostatic potential surfaces (ESP), and the higher electrostatic potential of the involved phenyl in TPA can be observed, compared to those in other D moieties. Accordingly, the strong conjugated effect in G‐TPA‐CH_3_ and G‐TPA results in the overlap of the lowest unoccupied molecular orbital (LUMO) and highest occupied molecular orbital (HOMO), while the charge separation can be detected in these luminogens with DMAC and PTZ as the D moieties (Figure 2c,d). For CS moiety, the additional sulfuryl with electronic‐withdrawing property, compared to other D moieties, can largely decrease the electron‐donating property, resulting in the weaker ICT effect with slightly blue‐shifted absorption wavelengths of G‐CS‐CH_3_ (476 nm) and G‐CS (474 nm). It can also be confirmed by the distribution of their HOMOs, which were mainly located at the BT skeletons, but much less on CS moiety.

All of these luminogens demonstrated bright red‐emission in THF solution at room temperature. Accompanying with the increased electron‐donating abilities of D moieties, the emission peaks of their photoluminescence (PL) spectra gradually red‐shifted from 586 nm (G‐CS‐CH_3_), to 594 nm (G‐DMAC‐CH_3_), then to 613 nm (G‐PTZ‐CH_3_), and finally to 625 nm (G‐TPA‐CH_3_) (Figure 2e; Table S1, Supporting Information). Similar PL spectra can be detected for G‐D series (Figure 2f), indicating that the ending groups (─COOCH_3_/─COOH) had little impact on their emission properties as isolated states. Once the temperature was cooled to 77 K, the obvious NIR phosphorescence can be detected with the onset wavelengths extended to 800 nm (Figure S2, Supporting Information). Among them, G‐CS‐CH_3_ and G‐CS exhibited the stronger phosphorescence intensity with the maximum phosphorescence wavelength of 689 and 690 nm, respectively.

When these luminogens aggregated into the solid states, the bright red fluorescence can still be observed (Figure S3, Supporting Information), with their photoluminescence quantum yields (PLQYs) ranging from 19% to 51% (Figure S4, Supporting Information). Compared to their fluorescence in solution as isolated states, the obvious red‐shifted emission can be detected (Figure 2g,h), mainly due to the strong intermolecular interactions at aggregated states. The G‐D series demonstrated a slightly red‐shifted emission than that of the corresponding G‐D‐CH_3_ ones (Table S1, Supporting Information). For instance, the maximum emission wavelength of G‐TPA‐CH_3_ was located at 652 nm, while that of G‐TPA was 665 nm. It may be related to the different molecular packing with the formation of hydrogen bonding by ─COOH moieties as ending groups. After removing the excitation source, the red/NIR afterglow can be recorded with the lasting time extended to 50 s (Figure S3, Supporting Information). Accordingly, the phosphorescence spectra of these luminogens at aggregated states demonstrated the two main emission peaks at ≈636–670 nm and 710–740 nm (Figure 2i,j). Among them, G‐TPA‐CH_3_ and G‐TPA with TPA as the D moiety exhibited the red‐shifted phosphorescence emission for the better‐conjugated effect and stronger ICT effect. While G‐CS‐CH_3_ and G‐CS demonstrated longer phosphorescence emission with RTP lifetime of 118 and 46 ms, respectively (Figure 2k,l; Figure S5, Supporting Information). The bright phosphorescence with persistent afterglow may be partially derived from the promoted intersystem crossing (ISC) process by the tunable electronic effect of different D moieties, as proved by the calculated spin‐orbit coupling (SOC) constants from singlet excited states to triplet excited states (Figure 2m). Among various ISC channels, S_1_→T_2_ and S_1_→T_3_ demonstrated the larger SOC values as the main ISC processes (Figure S6, Supporting Information). For G‐D‐CH_3_ series, the highest SOC values of S_1_→T_2_ and S_1_→T_3_ processes were achieved by G‐CS‐CH_3_ with 1.18 and 0.79 cm^‐1^, respectively (Figure 2n). Also, G‐CS exhibited the highest SOC values of 1.21 and 0.81 cm^−1^ for S_1_→T_2_ and S_1_→T_3_ in G‐D series with different D moieties, indicating CS moieties can be beneficial to the ISC process (Figure 2o). Also, the induced C─H…O and C─H…S interactions by the additional sulfuryl (O═S═O) moieties can suppress the possible nonradiative transitions, promoting to the generation of persistent red/NIR RTP property.

**Figure 3:**
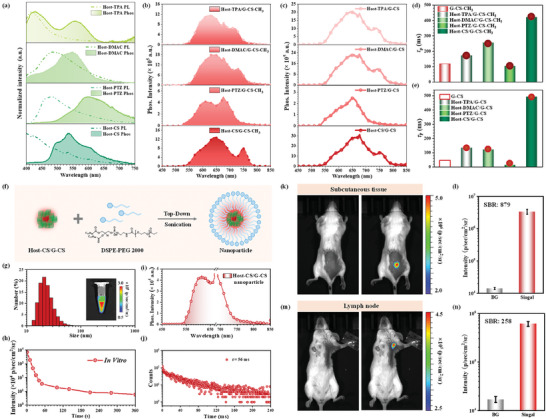
a) Normalized PL and phosphorescence spectra of Host‐D series at solid state. b) Phosphorescence spectra of Host‐D/G‐CS‐CH_3_ with a mass ratio of 10:1 at solid state. c) Phosphorescence spectra of Host‐D/G‐CS with mass ratio of 10:1 at solid state. d) RTP lifetimes of G‐CS‐CH_3_ and Host‐D/G‐CS‐CH_3_ systems at solid state. e) RTP lifetimes of G‐CS and Host‐D/G‐CS systems at solid state. f) Fabrication process of Host‐CS/G‐CS nanoparticles by top‐down approaches. g) Size distribution of Host‐CS/G‐CS nanoparticles. Insert: The afterglow image of Host‐CS/G‐CS nanoparticles taken by an IVIS instrument in bioluminescent mode, which was acquired at *t* = 10 s after removing white light. h) Time‐dependent afterglow decay of Host‐CS/G‐CS nanoparticles in vitro. i) Phosphorescence spectrum of Host‐CS/G‐CS nanoparticles in the aqueous solution. j) Time‐dependent phosphorescence decay of Host‐CS/G‐CS nanoparticles in the aqueous solution. k) White‐light‐excited afterglow bioimaging of the living mice with subcutaneous injection of Host‐CS/G‐CS nanoparticles, taken by an IVIS instrument in bioluminescent mode (*n* = 3). l) The quantification of afterglow intensity in bioimaging of subcutaneous tissue (*n* = 3; means±SD). m) White‐light‐excited afterglow bioimaging of lymph nodes in the living mice with an injection of Host‐CS/G‐CS nanoparticles, taken by an IVIS instrument in bioluminescent mode (*n* = 3). n) The quantification of afterglow intensity in bioimaging of lymph node (*n* = 3; means±SD).

As for host luminogens (Host‐D) with the D moieties as the RTP source and alkynyl as the ending groups, the obvious green or orange afterglow can be observed at crystalline states by naked eyes with lasting time in the range of 0.01–0.10 s (Figure S7, Supporting Information). Accordingly, the maximum emission wavelengths of phosphorescence spectra located at 560 nm for Host‐TPA, 550 nm for Host‐DMAC, 600 nm for Host‐PTZ, and 536 nm for Host‐CS, and their RTP lifetimes were in the region of 1.69–6.20 ms (**Figure** **3**a; Figure S8, Supporting Information). Compared to their phosphorescence spectra in the dilute solution as an isolated state at 77 K, the ≈100 nm red‐shifts were mainly due to the varied molecular conformations and intermolecular interactions at aggregated states, including C─H…π, C─H…O and π–π interactions (Figures S9 and S10, Supporting Information). The phosphorescence lifetime can increase up to 1.56 s for Host‐CS crystals at 77 K, mainly due to the efficient suppression of molecular motions as nonradiative transitions (Figure S11, Table S2, Supporting Information).

Accordingly, the PL and phosphorescence spectra of the Host‐D series and absorption of G‐D‐CH_3_ and G‐D are overlapped to ensure the multiple energy transfer in these host–guest systems, with Host‐D as hosts and G‐D‐CH_3_ or G‐D as guests (Figure S12, Supporting Information). With the aim to improve the RTP property of host–guest systems, the mass ratios (host/guest) between them were adjusted from 1:1 to 10000:1. Taking Host‐CS/G‐CS as an example, 10:1 of host/guest was determined as the optimized one for the brighter and red‐shifted phosphorescence emission, together with the prolonger lifetime (Figures S13 and S14, Supporting Information), which can be applied into another host–guest systems. Taking G‐CS‐CH_3_ and G‐CS with NIR phosphorescence and relatively longer RTP lifetimes in G‐D‐CH_3_ and G‐D series as the guests, the corresponding Host‐D/G‐CS‐CH_3_ and Host‐D/G‐CS systems demonstrated the obvious NIR phosphorescence and further prolonged RTP lifetimes (Figure 3b–e; Figure S15, Supporting Information). Especially for Host‐CS/G‐CS system, the phosphorescence lifetime can increase to 492 ms, 10.7‐times higher than that of G‐CS (46 ms). Furthermore, the efficiencies of energy transfer from hosts to guests have been calculated by the varied RTP lifetimes (Table S3, Supporting Information), and all of them exhibited over 99% efficiency, indicating the highly efficient energy transfer. The detailed processes can be investigated by the varied lifetimes of excited states under different conditions (Figure S16, Supporting Information). Accordingly, the phosphorescence lifetime@536 nm decreased from 1.69 ms (Host‐CS) to 14 µs (Host‐CS/G‐CS) with the doping process, while the fluorescence lifetimes@396 nm almost maintained with 1.5 ns (Host‐CS) and 1.8 ns (Host‐CS/G‐CS), respectively. It indicated that the energy transfer in Host‐CS/G‐CS system was mainly from the triplet excited state of Host‐CS. As to G‐CS as an energy acceptor, the phosphorescence lifetime@650 nm increased from 46 ms (G‐CS) to 492 ms (Host‐CS/G‐CS), indicating the possible triplet‐to‐triplet energy transfer (TTET) and triplet‐to‐singlet energy transfer (TSET) processes with the matched energy levels (Figure S17, Supporting Information) and the overlapped spectra. However, detailed changes in fluorescence lifetimes at red/NIR region could not be obtained, mainly due to the similar fluorescence (632 nm) and phosphorescence (650 nm) wavelengths. Thus, the highly efficient energy transfer was mainly related to the multiple channels, as well as the strong intermolecular interactions and optimized aggregated structures.

Considering the improved NIR phosphorescence property of Host‐CS/G‐CS system, the corresponding application in bioimaging has been conducted by the fabrication of Host‐CS/G‐CS nanoparticles (NPs) with a top‐down approach (Figure 3f). The average diameter of Host‐CS/G‐CS NPs was ≈55 nm, as measured by dynamic light scattering (DLS) (Figure 3g). More importantly, it can demonstrate the persistent NIR phosphorescence with a lifetime up to 56 ms (Figure 3i,j), with little quenching effect by oxygen and water, beneficial to the bio‐applications. Accordingly, in vivo imaging systems (IVIS) were utilized to collect the detected photon signals and record the optical images under the bioluminescence mode. After pre‐irradiation by white light, the afterglow signals of nanoparticles were as high as 3.694 × 10^7^ p sec^−1^ cm^2^ sr^−1^, and can last up to 5 min (Figure 3h). Subsequently, the ability of Host‐CS/G‐CS NPs for in vivo imaging was validated in living mice (Figure 3k‐n). Host‐CS/G‐CS NPs (1 mg mL^−1^, 50 µL) were subcutaneously injected into the back of the anesthetized living mice, afterglow images of the living mice were then captured after irradiation by the white lamp on the mice for 5 s. As demonstrated in Figure 3k, the mean afterglow intensity is 5.264 × 10^6^ p sec^−1^ cm^2^ sr^−1^, much higher than some of semiconducting polymer nanoparticles.^[^ ^11^ ^]^ The afterglow of these nanoparticles could avoid the biological auto‐fluorescence, thus displaying a low background noise with the intensity of 1.406 × 10^4^ p sec^−1^ cm^2^ s^−1^, and a high SBR of 879. Furthermore, with the aim to demonstrate the feasibility of these nanoparticles with red/NIR phosphorescence in the application of deep tissue penetration imaging, the utility of afterglow of Host‐CS/G‐CS nanoparticles was further evaluated for real‐time mapping of lymph nodes in living mice, since the lymph node mapping is clinically important in guiding surgical resection of tumor tissues.^[^ ^12^ ^]^ Accordingly, Host‐CS/G‐CS nanoparticles (1 mg mL^−1^, 50 µL) were intradermally injected into the forepaws of anesthetized living mice. At 5 h postinjection, the axillary lymph node could be clearly delineated with the afterglow of Host‐CS/G‐CS nanoparticles and can last for 280 s (Figure S18, Supporting Information). As shown in Figure 3m, the afterglow intensity of Host‐CS/G‐CS NPs is 6.119 × 10^5^ p sec^−1^ cm^2^ s^−1^, with the tissue background of 1.698 × 10^4^ p sec^−1^ cm^2^ s^−1^ under the same signal collection conditions. Therefore, the SBR for afterglow‐guided lymph node imaging by Host‐CS/G‐CS NPs is as high as 258, indicating the extremely low background noise and deep tissue penetration in in vivo tissue imaging.

**Figure 4:**
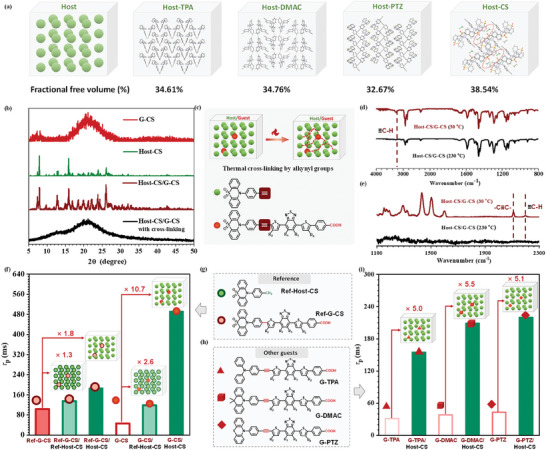
a) Molecular packing modes and fractional free volumes of Host‐TPA, Host‐DMAC, Host‐PTZ, and Host‐CS crystals. b) PXRD patterns of G‐CS, Host‐CS, and Host‐CS/G‐CS before and after thermal cross‐linking. c) Schematic diagram of thermal cross‐linking between alkynyl groups in host and guest molecules, and corresponding molecular structures. d) FTIR spectra of Host‐CS/G‐CS at 30 °C and after thermal annealing at 230 °C. e) Raman spectra of Host‐CS/G‐CS at 30 °C and after thermal annealing at 230 °C. f) RTP lifetimes of Ref‐G‐CS, Ref‐Host‐CS/Ref‐G‐CS, Host‐CS/Ref‐G‐CS, G‐CS, Ref‐Host‐CS/G‐CS, Host‐CS/G‐CS at solid states. (Insert: Schematic diagrams of guests and host–guest systems). g) Molecular structures of Ref‐Host‐CS and Ref‐G‐CS as the references. h) Molecular structures of G‐TPA, G‐DMAC, and G‐PTZ as the guests. i) RTP lifetimes of G‐TPA, G‐DMAC, and G‐PTZ at solid states as the guests, and the corresponding host–guest systems by the addition of Host‐CS as the host (Insert: Schematic diagrams of guests and host–guest systems).

The excellent NIR phosphorescence property of Host‐CS/G‐CS system and the large enhancement of RTP lifetime by the incorporation of host materials with visible phosphorescence emission inspire us to investigate the internal mechanism of these host–guest systems. Generally, host materials can afford the rigid environment by the crystalline matrix, which can largely suppress the nonradiative transitions of guest ones, promoting the NIR phosphorescence by the minimization of energy loss. In our case, Host‐D series demonstrated the ordered molecular packing at solid states, as demonstrated by the sharp peaks in powder X‐ray diffraction (PXRD) pattern (Figure S19, Supporting Information) and detailed crystal structures (**Figure** **4**a). The factional free volumes of these single crystals were 34.61% for Host‐TPA, 34.76% for Host‐DMAC, 32.67% for Host‐PTZ, and 38.54% for Host‐CS, the trend of which was in consistence with their crystal densities (Table S4, Supporting Information). The compact molecular packing was usually beneficial to the RTP emission for the restriction of molecular motions. Thus, the longest RTP lifetime of Host‐D series was achieved by Host‐PTZ with the smallest factional free volumes. However, after the incorporation of G‐CS as the guest, the resultant Host‐PTZ/G‐CS demonstrated the varied PXRD with shifts and the absence of some diffraction peaks, compared to that of Host‐PTZ, indicating the changed aggregated structures (Figure S19, Supporting Information). A similar phenomenon can also be found in Host‐TPA/G‐CS and Host‐DMAC/G‐CS systems, supporting that the addition of G‐CS can partially destroy the molecular packing of original host materials (Figure S19, Supporting Information). Interestingly, the diffraction peaks of Host‐CS and Host‐CS/G‐CS were almost the same, meaning the nearly undisturbed aggregated structures (Figure 4b). It may be related to the relatively loose molecular packing of Host‐CS crystal with large space (red circles in Figure 4a) among the layer‐by‐layer modes. The space may provide accommodation for guest molecules, favorable to their intermolecular interactions and energy transfer processes for the close distance. Since both host and guest molecules contained the alkynyl moieties as the reaction sites for thermal crosslinking, PXRD pattern of Host‐CS/G‐CS system after thermal annealing at 230° was conducted. As shown in Figure 4b, the transformation from crystalline to amorphous state can be detected. Meanwhile, the characterization peak of ≡C─H disappeared in Fourier transform infrared (FTIR) spectra (Figure 4d), and characterization peaks of C≡C and ≡C─H disappeared in Raman spectra (Figure 4e), indicating the thermal cross‐linking of C≡C moieties in Host‐CS and G‐CS molecules (Figure 4c; Figure S20, Supporting Information). It can further confirm the close distances of host and guest molecules in Host‐CS/G‐CS system, resulting in the efficient suppression of non‐radiative transitions of G‐CS as NIR phosphorescence source.

To further investigate the role of alkynyl moieties in the host and guest molecules, the reference molecules of Host‐CS and G‐CS by replacing C≡C with C─C were synthesized as Ref‐Host‐CS and Ref‐G‐CS, respectively (Figure 4g). Ref‐Host‐CS at crystalline state also demonstrated the green phosphorescence with the emission peak located at 500 nm (Figure S21, Supporting Information), and the corresponding lifetime can achieve up to 33 ms, much higher than that of Host‐CS (2.2 ms). It was also related to the compact molecular packing with strong intermolecular interactions in the single crystal. For Ref‐G‐CS, a similar red/NIR phosphorescence to that of G‐CS can be detected, with slight blue‐shifted absorption and emission spectra (Figures S22 and S23, Supporting Information). It was mainly due to the relatively twisted configuration with weaker ICT and lower dipole moment (Figure S23, Supporting Information). The RTP lifetime was as high as 103 ms at aggregated state. Furthermore, with the investigation of host–guest systems constructed by these luminogens with or without C≡C moiety, the corresponding mixed systems were conducted by Host‐CS or Ref‐Host‐CS as the host with/without terminal alkyne, G‐CS or Ref‐G‐CS as the guest with/without an alkynyl bridge (Figure 4f,g). For Ref‐G‐CS without alkynyl bridge as the guest, the resultant Ref‐Host‐CS/Ref‐G‐CS and Host‐CS/Ref‐G‐CS demonstrated the prolonged RTP lifetimes, further confirming the advantage of host–guest systems with visible and NIR phosphorescence materials. The ratios of RTP lifetimes from host–guest system and guest were 1.3 (Ref‐Host‐CS/Ref‐G‐CS) and 1.8 (Host‐CS/Ref‐G‐CS), respectively, the longer RTP lifetime of Host‐CS/Ref‐G‐CS indicated that the Host‐CS with terminal alkyne was the preferred host. For G‐CS with alkynyl bridge as the guest, the larger enhancement of RTP lifetime in Ref‐Host‐CS/G‐CS and Host‐CS/G‐CS can be detected with 2.6 and 10.7 times, respectively. The preferred host of Host‐CS was further confirmed, which may be related to the maintained crystal structures after the addition of Ref‐G‐CS and G‐CS as the guests. Under similar conditions, the PXRD patterns of Ref‐Host‐CS changed, indicating the partially destroyed crystal structures (Figure S24, Supporting Information). The difference between Host‐CS and Ref‐Host‐CS at aggregated states can be further explained by their detailed crystal structures. Although both Host‐CS and Ref‐Host‐CS crystals demonstrated the layer‐by‐layer packing modes, the interspacing varied much with 2.198 Å (Host‐CS) and 0.609 Å (Ref‐Host‐CS), respectively (Figure S25, Supporting Information). The large interspacing by Host‐CS can be beneficial to the incorporation of guest molecules with rod‐like sharpness, as further proved by the RTP property of Host‐CS/G‐CS and Ref‐Host‐CS/G‐CS under different atmospheres (Figures S26 and S27, Supporting Information). Under the vacuum or nitrogen atmosphere, the RTP property of Host‐CS/G‐CS remained the same with the unchanged phosphorescence spectra and lifetimes, compared to those under air conditions, indicating that the whole host–guest system was compact with nearly no quenching effect by oxygen and moisture. However, the RTP property of Ref‐Host‐CS/G‐CS exhibited the obviously improved RTP property with the increased phosphorescence intensity and prolonged lifetime (≈1.16‐folds). It meant that Ref‐Host‐CS and G‐CS at aggregated states were not well‐combined, which may be related to the too‐compact packing mode of Ref‐Host‐CS with no enough space to incorporate the guest molecules, resulting in the partially destroyed crystal structures as mentioned above. Considering the excellent aggregated structure of Host‐CS with visible RTP emission, various guests with NIR phosphorescence have been incorporated to verify the universality (Figure 4h). Excitingly, all the host–guest systems constructed by Host‐CS/G‐TPA, Host‐CS/G‐DMAC, and Host‐CS/G‐PTZ exhibited the much improved RTP property with lifetime increased by 5.0‐folds, 5.5‐folds, and 5.1‐folds, respectively, compared to those of guests themselves (Figure 4i; Figure S28, Supporting Information). Thus, the efficient way to achieve persistent NIR phosphorescence has been built by host–guest systems constructed by visible (host) and NIR (guest) phosphorescence materials, and the optimized strategies, apart from the matched energy levels, can be concluded as follows:

1. The host materials with rigid crystalline states are preferred, and the aggregated structures with enough space to accommodate the guest molecules should be more favorable, which can maintain the crystalline states of host–guest molecules, and promote the intermolecular interactions between host and guest molecules.
2. The ending groups of guest molecules play a key role in the host–guest systems and the formation of hydrogen bonding between them can favor suppressing the severe non‐radiative transitions of guest molecules, resulting in prolonged RTP emission.
3. The molecular configuration and electronic‐donating property of D moieties in guest molecules can well‐adjust ISC process by the tunable ICT effect, and the moderated electron donors with multiple interaction sites are preferred.

Thus, the persistent NIR phosphorescence has been achieved by the host and guest RTP materials with “1+1≻2” effect, mainly due to matched spatial occupation and strong intermolecular interactions, as well as the highly efficient energy transfers with multiple channels. It can be further optimized by the well‐tuned cavities in host materials and sizes of guests, and the incorporation of more kinds of strong intermolecular interactions with adjustable directions in the near further.

## 3 Conclusion

In summary, the host–guest systems with visible (host) and NIR (guest) phosphorescence materials have been constructed, to create strong NIR RTP with much higher performance than that of the original guests, for the first time. The host materials with rigid aggregated structures can efficiently suppress the severe nonradiative transitions of guests by strong intermolecular interactions and space restriction. Moreover, the highly efficient phosphorescence energy transfer can further improve the phosphorescence emission of guests, resulting in persistent NIR phosphorescence with ten‐folds enhancement of RTP lifetimes. The universality and priority of these host–guest systems have been proved by various luminogens with different donor groups and ending groups. Accordingly, the formation of hydrogen bonding between them, and the suitable space in host materials to accommodate guests are the preferred factors to prolong the lifetime of NIR phosphorescence, promoting their application in afterglow imaging with deeper penetration and higher SBRs. Thus, for the construction of host–guest RTP materials, the matched space restriction provided by the host to the guest should be considered seriously, in addition to the well‐recognized efficient energy transfer.

## 4 Experimental Section

### Materials

Unless otherwise stated, all starting materials and reagents were purchased from commercial suppliers and used without further purification. The solvents were carefully dried and distilled from appropriate drying agents before use. The Syntheses of target compounds were performed as Schemes S1 and S2 (Supporting Information), and characterized by ^1^H NMR, ^13^C NMR, and MS (EI or MALDI‐TOF).

### Theoretical Calculations

TD‐DFT calculations were performed on the Gaussian 09 program (Revision D.01). The ground state geometries were optimized using the PBE0 functional with def2svp basis. The ESP surfaces were obtained from Gaussian View 6 with an electron density isosurface value of 0.001. The excitation energies in the n‐th singlet and n‐th triplet excited states in the isolated state were obtained using the PBE0 functional with def2svp basis. The SOC values were performed through the same function and basis using PySOC package with python 2.7. To simplify the calculation process, the alkoxy substituents with long chains (‐OC_12_H_25_) were simplified to methoxy substituents.

### Preparation of Nanoparticles

DSPE‐PEG 2000 (10 mg), G‐CS (1 mg), and Host‐CS (10 mg) were dissolved in THF (2 mL), and then THF was evaporated with a gentle nitrogen flow. Then, deionized water (2 mL) was added, and the mixture was sonicated using a microtip‐equipped probe ultrasonic instrument (Scientz‐IID) for 20 min (duty cycle of 80%) to afford the NP solution. The aqueous solution was filtered through a PES syringe‐driven filter (0.22 µm) to afford the nanoparticles.

### In Vivo Afterglow Subcutaneous Imaging

Nanoparticles (1 mg mL^−1^, 50 µL) were subcutaneously injected into the right back of the anesthetized living mice. Afterglow images of the living mice were then captured on IVIS Spectrum System under bioluminescence mode (open filter; exposure time: 10 s) instantly after irradiation of the mice with white light (100 mW cm^−2^) for 5 s.

### In Vivo Afterglow Lymph Node Imaging

Nanoparticles (1 mg mL^−1^, 50 µL) were injected into the forepaws of anesthetized living mice. At 5 h postinjection, afterglow images of living mice were captured on IVIS Spectrum System under bioluminescence mode (open filter; exposure time: 10 s) immediately after irradiation with white light (100 mW cm^−2^) for 5 s.

### Statistical Analysis

The in vivo phosphorescence signals were quantified with region of interest analysis using Living Image 4.7.2 software (PerkinElmer Inc.), which were repeated independently three times. Results are expressed as the mean ± SD. The ratios of the standard deviation to the mean values were smaller than 5%. All statistical calculations were performed using the Origin 2022 learning edition (OriginLab Corporation).

### Statement of Ethical Approval

All animal studies were performed according to the guidelines, and the overall project protocols were approved by the Welfare and Ethics Committee of Laboratory Animal, College of Life Sciences, Wuhan University (approval no. WDSKY0202101).

## Conflict of Interest

The authors declare no conflict of interest.

## Supporting information

Supporting Information

## Data Availability

Research data are not shared.

## References

[1] a) R. Kabe , C. Adachi , Nature 2017, 550, 384; 28967911 10.1038/nature24010

[2] a) B. Chang , J. Chen , J. Bao , T. Sun , Z. Cheng , Chem. Rev. 2023, 123, 13966; 37991875 10.1021/acs.chemrev.3c00401

[3] a) L. Flamigni , A. Barbieri , C. Sabatini , B. Ventura , F. Barigelletti , in Photochemistry and Photophysics of Coordination Compounds II (Eds: V. Balzani , S. Campagna ), Springer Berlin Heidelberg, Berlin, Heidelberg 2007, pp. 143;

[4] a) X. Chen , C. Xu , T. Wang , C. Zhou , J. Du , Z. Wang , H. Xu , T. Xie , G. Bi , J. Jiang , X. Zhang , J. N. Demas , C. O. Trindle , Y. Luo , G. Zhang , Angew. Chem. Int. Ed. 2016, 55, 9872; 10.1002/anie.201601252 27385550

[5] a) S. Xu , R. Chen , C. Zheng , W. Huang , Adv. Mater. 2016, 28, 9920; 27634285 10.1002/adma.201602604

[6] a) E. Gabellieri , G. B. Strambini , A. Baracca , G. Solaini , Biophys. J. 1997, 72, 1818; 9083686 10.1016/S0006-3495(97)78828-3 PMC1184376

[7] a) R. Gao , Y. Cha , H. M. Ahmad , H. Fu , Z. Yu , Adv. Opt. Mater. 2023, 11, 2301112;

[8] a) F. Lin , H. Wang , Y. Cao , R. Yu , G. Liang , H. Huang , Y. Mu , Z. Yang , Z. Chi , Adv. Mater. 2022, 34, 2108333; 10.1002/adma.202108333 35137460

[9] a) Y. Lei , W. Dai , J. Guan , S. Guo , F. Ren , Y. Zhou , J. Shi , B. Tong , Z. Cai , J. Zheng , Y. Dong , Angew. Chem. Int. Ed. 2020, 59, 16054; 10.1002/anie.202003585 32500576

[10] a) M. J. Frisch , G. W. Trucks , H. B. Schlegel , G. E. Scuseria , M. A. Robb , J. R. Cheeseman , G. Scalmani , V. Barone , B. Mennucci , G. A. Petersson , H. Nakatsuji , M. Caricato , X. Li , H. P. Hratchian , A. F. Izmaylov , J. Bloino , G. Zheng , J. L. Sonnenberg , M. Hada , M. Ehara , K. Toyota , R. Fukuda , J. Hasegawa , M. Ishida , T. Nakajima , Y. Honda , O. Kitao , H. Nakai , T. Vreven , J. A. Montgomery Jr. , et al., Gaussian09, Revision D.01 and Gaussian16, Revision C.01, Gaussian, Inc, Wallingford CT, 2019;

[11] a) J. Huang , K. Pu , Angew. Chem. Int. Ed. 2020, 59, 11717; 10.1002/anie.202001783 32134156

[12] a) A. K. Polomska , S. T. Proulx , Adv. Drug Deliv. Rev. 2021, 170, 294; 32891679 10.1016/j.addr.2020.08.013

