# Supplementary material for: Excellent Persistent Near‐Infrared Room Temperature Phosphorescence from Highly Efficient Host–Guest Systems
Source: Adv Sci (Weinh). 2024 May 17;11(28):2402846. doi: 10.1002/advs.202402846 (PMC11267349; doi:10.1002/advs.202402846)
Supplement: Supplementary file 1 — Supporting Information [file ADVS-11-2402846-s001.docx]

Supporting Information

Excellent Persistent Near-infrared Room Temperature Phosphorescence from Highly Efficient Host-Guest Systems

Shuhui Li, Juqing Gu, Jiaqiang Wang, Wentao Yuan, Guigui Ye, Likai Yuan, Qiuyan Liao, Le Wang, Zhen Li*, and Qianqian Li*

**Synthesis and characterizations.**

**Materials.**

4-Bromo-*N*,*N*-diphenylaniline, 9,10-dihydro-9,9-dimethylacridine, 10H-phenothiazine, 1-bromo-4-iodobenzene, 4,7-dibromo-5,6-bis(dodecyloxy)benzo[*c*][1,2,5] thiadiazole, 3-(2-ethylhexyl) thiophene, chlorotributyltin, ethynyltrimethylsilane (TMSA), copper (I) iodide (CuI), *trans*-dichlorobis(triphenyl-phosphine)palladium(II) (Pd(PPh_3_)_2_Cl_2_), bis[di-*tert*-butyl-(4-dimethylamino phenyl)phosphine]dichloropalladium(II) (Pd(Amphos)_2_Cl_2_), palladium (Ⅱ) acetate (Pd(OAc)_2_), triphenylphosphine (PPh_3_), potassium *tert*-butanolate (*t*-BuOK), tri-*tert*-butylphosphine (P(*t*-Bu)_3_), diisopropylamine (DIPA), potassium carbonate (K_2_CO_3_), *N*-bromosuccinimide (NBS), butyllithium (BuLi), triethylamine (TEA), methanol (MeOH), acetic acid (AcOH), hydrogen peroxide (H_2_O_2_), dichloromethane (DCM), chloroform (CHCl_3_), petroleum ether (PE) were commercial source and directly used without further purification. The toluene (Tol) and tetrahydrofuran (THF) (chromatographic pure) were taken from the anhydrous and anaerobic system of Vigor company.

**Characterization.**

^1^H NMR and ^13^C NMR spectra were measured by Bruker AVANCE 400 MHz spectrometer or JNM-ECZ600R/S1 (tetramethylsilane as the internal standard). Mass spectra (EI) were conducted on a Shimadzu GCMS-QP2020. Matrix-assisted laser desorption ionization time-of-flight (MALDI-TOF) mass spectra were recorded on an AB SCIEX MALDI-TOF/TOF 5800. Single-crystal X-ray diffraction data of these samples were collected at room temperature on a SuperNova, Dual, Cu at zero, atlas diffractometer equipped with graphite-monochromated Cu Kα radiation (*λ* = 1.54184 Å). UV-Vis absorption spectra were obtained on a Shimadzu UV-2700 spectrophotometer at room temperature. The photoluminescence (PL) spectra and phosphorescence spectra of samples in solutions at room temperature and at 77K were measured by Hitachi F-4700. The PL spectra, phosphorescence spectra, time-resolved phosphorescence decay curves and photoluminescence quantum yields (PLQY) of samples as solid states were measured on Edinburgh Analytical instrument FLS 980 equipped with both xenon arc lamp (450 W) and pulsed flash lamps. The time-resolved phosphorescence-decay curves in the NIR region were measured on a PicoQuant FluoTime 300. Fourier transform infrared (FT-IR) spectra and Raman spectra were measured with FT-IR Nicolet iS10 and XploRA Plus Raman spectrometer, respectively. The powder X-ray diffraction (PXRD) patterns were recorded by Rigaku MiniFlex 600 with an X-ray source of Cu K*α* (*λ* = 1.5418 Å) at 25 ^o^C at 40 KV and 15 mA at a scan rate of 10^o^ (2*θ*)/min (scan range: 5-50^o^). Afterglow photographs were performed by multi-photoluminescence imager, Tanon-5200 multi. Dynamic light scattering (DLS) was monitored on a Malvern Nano-ZS90 Particle Sizer. *In vitro* and *in vivo* phosphorescence imaging were conducted on *in vivo* imaging systems (IVIS) Spectrum imaging system (PerkinElmer Inc.; the cooling temperature of charge-coupled device lens is −85°C).

**Synthetic Routes:**

**Scheme S1.** Synthetic routes of **Host-D**. Reagents and conditions: i) Pd(OAc)_2_, P(*t*-Bu)_3_, *t*-BuOK, Tol, 110 ^o^C; ii) H_2_O_2_, AcOH, DCM, 60 ^o^C; iii) TMSA, PdCl_2_(PPh_3_)_2_, PPh_3_, CuI, DIPA, 80 ^o^C; iv) K_2_CO_3_, THF, MeOH, 25 ^o^C.

**The general synthesis of compound DMAC-Br and PTZ-Br:**

Under the atmosphere of nitrogen, the mixture of 1-bromo-4-iodobenzene (1.20 equiv.), 9,9-dimethyl-9,10-dihydroacridine or 10H-phenothiazine (1.00 equiv.), *t*-BuOK (3.00 equiv.), Pd(OAc)_2_ (0.05 equiv.), and P(*t*-Bu)_3_ (0.08 equiv.) in anhydrous toluene (30 mL) was placed in a Schlenk tube and stirred at 110 ^o^C for 6 h. After being cooled to room-temperature, the mixture was poured into water and extracted with dichloromethane for three times. The organic phase was evaporated under vacuum and purified by column chromatography on silica gel.

**DMAC-Br:** White solid (470 mg, 26%). ^1^H NMR (400 MHz, CDCl_3_) δ (ppm): 7.76 (d, *J* = 8.5 Hz, 2H, ArH), 7.46 (d, *J* = 7.4 Hz, 2H, ArH), 7.23 (d, *J* = 8.5 Hz, 2H, ArH), 7.02 – 6.90 (m, 4H, ArH), 6.25 (d, *J* = 8.7 Hz, 2H, ArH), 1.68 (s, 6H, CH_3_). ^13^C NMR (100 MHz, CDCl_3_) δ (ppm): 140.60, 140.31, 134.23, 133.24, 130.10, 126.43, 125.34, 122.11, 120.82, 113.90, 35.98, 31.25.

**PTZ-Br:** White solid (1.63 g, 46%). ^1^H NMR (400 MHz, CDCl_3_) δ (ppm): 7.71 (d, *J* = 8.6 Hz, 2H, ArH), 7.29 – 7.25 (m, 2H, ArH), 7.06 (d, *J* = 7.3 Hz, 2H, ArH), 6.92 – 6.82 (m, 4H, ArH), 6.25 (d, *J* = 7.9 Hz, 2H, ArH). ^13^C NMR (100 MHz, CDCl_3_) δ (ppm): 143.80, 140.34, 133.98, 132.01, 126.93, 122.92, 121.64, 121.10, 116.52.

**Synthesis of CS-Br**

**PTZ-Br** (1.06 g, 3.00 mmol) in AcOH (24 mL) was placed in a round-bottom flask and stirred for half an hour. 30% H_2_O_2_ (36 mL) was added to the mixture dropwise at room temperature. After being refluxed for 10 h, the mixture was cooled to room temperature and filtered. The white solid was washed with water until pH value near to 7, and dried in vacuum oven to yield a white solid (1.06 g, 92%). ^1^H NMR (400 MHz, CDCl_3_) δ (ppm): 8.17 (dd, *J* = 7.9, 1.5 Hz, 2H, ArH), 7.89 – 7.79 (m, 2H, ArH), 7.41 (m, 2H, ArH), 7.31 – 7.26 (m, 4H, ArH), 6.62 (d, *J* = 8.6 Hz, 2H, ArH). ^13^C NMR (100 MHz, CDCl_3_) δ (ppm): 140.50, 137.90, 134.73, 132.89, 132.32, 123.95, 123.58, 122.87, 122.34, 116.99.

**The general synthesis of compound Host-D:**

Under the atmosphere of nitrogen, the mixture of bromobenzene derivatives with various electronic donors (D-Br) (1.00 equiv.), CuI (0.03 equiv.), Pd(PPh_3_)_2_Cl_2_ (0.05 equiv.), PPh_3_ (0.03 equiv.) and TMSA (2.00 equiv.) in DIPA (10 mL) was placed in a Schlenk tube and stirred at 80 ^o^C for 5 h. After being cooled to room-temperature, the mixture was filtered. The filtrate was washed with a saturated ammonium chloride solution and extracted by dichloromethane for three times. After the solvent was removed under vacuum, the mixture was purified by column chromatography on silica gel and used directly in the next step.

The mixture of one of the above compounds (1.00 equiv.), K_2_CO_3_ (10.00 equiv.), THF (6 mL), and MeOH (6 mL) was placed in a round-bottom flask and stirred at room temperature for 2 h. Then, the mixture was washed with water and extracted by dichloromethane for three times. The organic phase was evaporated under vacuum and purified by column chromatography on silica gel to afford Host-D.

**Host-TPA**: White solid (175 mg, 65%). ^1^H NMR (400 MHz, CDCl_3_) δ (ppm): 7.34 (d, *J* = 8.0 Hz, 2H, ArH), 7.27 (d, *J* = 6.6 Hz, 4H, ArH), 7.08 (*m*, 6H, ArH), 6.97 (d, *J* = 7.9 Hz, 2H, ArH), 3.03 (s, 1H, ≡CH). ^13^C NMR (100 MHz, CDCl_3_) δ (ppm): 148.37, 147.13, 133.08, 129.43, 125.06, 123.65, 122.06, 114.75, 83.94, 76.19. MS (EI, *m/z*): [M]^+^ calcd. for C_20_H_15_N, 269.12; found, 269.20.

**Host-DMAC**: White solid (200 mg, 65%). ^1^H NMR (400 MHz, CDCl_3_) δ (ppm): 7.78 (d, *J* = 8.2 Hz, 2H, ArH), 7.49 (d, *J* = 8.7 Hz, 2H, ArH), 7.36 (d, *J* = 3.6 Hz, 2H, ArH), 7.04 – 6.93 (m, 4H, ArH), 6.29 (d, *J* = 8.0 Hz, 2H, ArH), 3.21 (s, 1H, ≡CH), 1.72 (s, 6H, CH_3_). ^13^C NMR (100 MHz, CDCl_3_) δ (ppm): 141.77, 140.63, 134.75, 133.89, 133.70, 131.51, 130.15, 128.76, 128.58, 128.51, 126.45, 125.35, 122.18, 120.84, 114.02, 82.96, 78.44, 36.02, 31.27. MS (EI, *m/z*): [M]^+^ calcd. for C_23_H_19_N, 309.15; found, 309.25.

**Host-PTZ**: Yellow solid (280 mg, 79%). ^1^H NMR (400 MHz, CDCl_3_) δ (ppm): 7.67 (d, *J* = 8.3 Hz, 2H, ArH), 7.32 (s, 2H, ArH), 7.11 (d, *J* = 7.4 Hz, 2H, ArH), 6.98 – 6.87 (m, 4H, ArH), 6.40 (d, *J* = 8.2 Hz, 2H, ArH), 3.17 (s, 1H, ≡CH). ^13^C NMR (100 MHz, CDCl_3_) δ (ppm): 143.56, 142.22, 137.27, 137.16, 134.39, 133.88, 133.69, 128.76, 128.57, 128.53, 128.51, 127.19, 126.98, 123.27, 122.61, 120.90, 117.79, 82.99, 78.17. MS (EI, *m/z*): [M]^+^ calcd. for C_20_H_13_NS, 299.08; found, 299.15.

**Host-CS**: White solid (930 mg, 94%). ^1^H NMR (400 MHz, CDCl_3_) δ (ppm): 8.17 (dd, *J* = 7.9, 1.5 Hz, 2H, ArH), 7.81 (d, *J* = 8.4 Hz, 2H, ArH), 7.43 – 7.34 (m, 4H, ArH), 7.28 (s, 2H, ArH), 6.61 (d, *J* = 8.6 Hz, 2H, ArH), 3.26 (s, 1H, ≡CH). ^13^C NMR (100 MHz, CDCl_3_) δ (ppm): 140.50, 139.08, 135.07, 132.87, 130.71, 124.01, 123.55, 122.80, 122.29, 117.04, 82.14, 79.59. MS (EI, *m/z*): [M]^+^ calcd. for C_20_H_13_NO_2_S, 331.07; found, 331.15.

**Scheme S2.** Synthetic routes of **G-D-CH_3_** and **G-D**. Reagents and conditions: i) *n*-BuLi, THF, -78 ^o^C to 25 ^o^C; ii) Pd(PPh_3_)_2_Cl_2_, THF, 80 ^o^C; iii) CHCl_3_, AcOH, 25 ^o^C; iv) CuI, PdCl_2_(PPh_3_)_2_, THF, Et_3_N, 70 ^o^C; v) Pd(amphos)Cl_2_, K_2_CO_3_, THF, H_2_O, 80 ^o^C; vi) KOH, H_2_O, THF, 80 ^o^C.

**Synthesis of ThSn**

Under the atmosphere of nitrogen, 3-(2-ethylhexyl)thiophene (2.94 g, 15.00 mmol) in anhydrous THF (40 mL) was placed in a Schlenk tube and stirred at -78 °C. *n*-Butyllithium (6 mL, 11.00 mmol) was added dropwise, and the solution was stirred for 1.5 h under the same temperature. Then tributyltin chloride (4.5 mL) was added, and the reaction mixture was recovered to room temperature and stirred overnight. The mixture was poured into water and extracted with dichloromethane for three times. The solvent was removed under vacuum, and the product was used directly in the next step without further purification.

**Synthesis of BT**

Under the atmosphere of nitrogen, the mixture of 4,7-dibromo-5,6-bis(dodecyloxy) benzo[*c*][1,2,5]thiadiazole (3.96 g, 6.00 mmol), Pd(PPh_3_)_2_Cl_2_ (462 mg, 0.40 mmol), and ThSn in anhydrous THF was placed in a Schlenk tube and stirred at 80 ^o^C for 8 h. After being cooled to room-temperature, the mixture was poured into water and extracted by dichloromethane for three times. The organic phase was evaporated under vacuum and purified by column chromatography on silica gel to yield **BT** as an orange solid (2.24 g, 42%). ^1^H NMR (400 MHz, CDCl_3_) δ (ppm): 8.29 (s, 2H, ArH), 7.07 (s, 2H, ArH), 4.10 (t, *J* = 7.1 Hz, 4H, OCH_2_), 2.65 (d, *J* = 6.9 Hz, 4H, CH_2_), 1.97 – 1.87 (m, 4H, CH_2_), 1.65 (m, 4H, CH, CH_2_), 1.43 (d, *J* = 7.7 Hz, 4H, CH_2_), 1.37 (s, 2H, CH_2_), 1.30 (m, 44H, CH_2_), 0.94–0.88 (m, 18H, CH_3_). ^13^C NMR (100 MHz, CDCl_3_) δ (ppm): 151.94, 151.02, 141.61, 133.62, 132.48, 123.22, 117.59, 74.23, 40.40, 34.66, 32.55, 31.95, 30.39, 29.74, 29.68, 29.64, 29.40, 28.94, 26.02, 25.69, 23.12, 22.72, 14.20, 14.14, 10.90.

**Synthesis of 2Br-BT**

The mixture of BT (1.79 g, 2.00 mmol), NBS (712 mg, 4.00 mmol) in the solution (trichloromethane: acetic acid = 1:1) was placed in a round-bottom flask and stirred at room temperature for 3 h in the dark. The mixture was poured into water and extracted by dichloromethane for three times. After the solvent was removed under vacuum, the mixture was purified by column chromatography on silica gel to yield **2Br-BT** as a yellow solid (988 mg, 47%). ^1^H NMR (400 MHz, CDCl_3_) δ (ppm): 8.29 (s, 2H, ArH), 4.12 (t, *J* = 7.2 Hz, 4H, OCH_2_), 2.60 (d, *J* = 7.2 Hz, 4H, CH_2_), 2.01 – 1.87 (m, 4H, CH_2_), 1.79 – 1.67 (m, 2H, CH), 1.49 – 1.22 (m, 52H, CH_2_), 0.99 – 0.80 (m, 18H, CH_3_). ^13^C NMR (100 MHz, CDCl_3_) δ (ppm): 151.55, 150.51, 140.93, 133.57, 132.18, 129.37, 125.13, 124.42, 117.02, 123.23, 116.94, 113.25, 74.47, 40.02, 33.86, 32.53, 31.96, 30.33, 29.74, 29.70, 29.59, 28.81, 25.98, 25.75, 23.13, 22.72, 14.18, 10.90.

**The general synthesis of compound** **G-D-Br:**

Under the atmosphere of nitrogen, the mixture of Host-D (2.50 equiv.), 2Br-BT (1.00 equiv.), CuI (0.20 equiv.), Pd(PPh_3_)_2_Cl_2_ (0.10 equiv.) in deaerating anhydrous THF (15 mL) and triethylamine (8 mL) was placed in a Schlenk tube and stirred at 70 ^o^C for 7 h. After being cooled, the mixture was poured into water and extracted with dichloromethane for three times. The organic phase was evaporated under vacuum and purified by column chromatography on silica gel.

**G-TPA-Br**: Red solid (170 mg, 9.2%). ^1^H NMR (400 MHz, CDCl_3_) δ (ppm): 8.34 (m, 2H, ArH), 7.38 (d, *J* = 8.7 Hz, 2H, ArH), 7.32 – 7.27 (m, 4H, ArH), 7.13 (d, *J* = 7.5 Hz, 4H, ArH), 7.09 – 7.01 (m, 4H, ArH), 4.13 (q, *J* = 7.3 Hz, 4H, OCH_2_), 2.78 (d, *J* = 7.0 Hz, 2H, CH_2_), 2.61 (d, *J* = 7.2 Hz, 2H, CH_2_), 1.96 (p, *J* = 7.2 Hz, 4H, CH_2_), 1.77 (m, 2H, CH), 1.41 – 1.19 (m, 52H, CH_2_), 0.96 – 0.85 (m, 18H, CH_3_).

**G-DMAC-Br**: Red solid (59 mg, 31%). ^1^H NMR (400 MHz, CDCl_3_) δ (ppm): 8.43 (s, 1H, ArH), 8.33 (s, 1H, ArH), 7.80 (d, *J* = 8.4 Hz, 2H, ArH), 7.48 (dd, *J* = 7.6, 1.7 Hz, 2H, ArH), 7.36 (d, *J* = 8.4 Hz, 2H, ArH), 7.03– 6.94 (m, 4H, ArH), 6.33 (dd, *J* = 8.0, 1.3 Hz, 2H, ArH), 4.16 (t, *J* = 7.2 Hz, 4H, OCH_2_), 2.85 (dd, *J* = 7.0, 2.8 Hz, 2H, CH_2_), 2.62 (d, *J* = 7.2 Hz, 2H, CH_2_), 2.04 – 1.93 (m, 4H, CH_2_), 1.86 (d, *J* = 4.5 Hz, 1H, CH), 1.79 – 1.73 (m, 1H, CH), 1.71 (s, 6H, CH_3_), 1.48 (d, *J* = 6.8 Hz, 4H, CH_2_), 1.33 (m, 48H, CH_2_), 1.03 – 0.87 (m, 18H, CH_3_).

**G-PTZ-Br**: Red solid (42 mg, 11%). ^1^H NMR (400 MHz, CDCl_3_) δ (ppm): 8.40 (s, 1H, ArH), 8.32 (s, 1H, ArH), 7.71 (d, *J* = 8.5 Hz, 2H, ArH), 7.35 (d, *J* = 8.5 Hz, 2H, ArH), 7.09 (d, *J* = 9.1 Hz, 2H, ArH), 6.96 – 6.84 (m, 4H, ArH), 6.39 (d, *J* = 8.0 Hz, 2H, ArH), 4.14 (t, *J* = 7.1 Hz, 4H, OCH_2_), 2.82 (d, *J* = 7.3 Hz, 2H, CH_2_), 2.61 (d, *J* = 7.2 Hz, 2H, CH_2_), 2.05 – 1.90 (m, 4H, CH_2_), 1.78 (m, 2H, CH), 1.54 – 1.18 (m, 52H, CH_2_), 1.04 – 0.81 (m, 18H, CH_3_).

**G-CS-Br**: Red solid (142 mg, 12%). ^1^H NMR (400 MHz, CDCl_3_) δ (ppm): 8.41 (s, 1H, ArH), 8.32 (s, 1H, ArH), 8.19 (dd, *J* = 7.9, 1.5 Hz, 2H, ArH), 7.84 (d, *J* = 8.4 Hz, 2H, ArH), 7.40 (dd, *J* = 7.7, 5.1 Hz, 4H, ArH), 7.28 (d, *J* = 7.9 Hz, 2H, ArH), 6.68 (d, *J* = 8.6 Hz, 2H, ArH), 4.19 – 4.11 (m, 4H, OCH_2_), 2.84 (d, *J* = 8.4 Hz, 2H, CH_2_), 2.61 (d, *J* = 7.2 Hz, 2H, CH_2_), 1.96 (m, 4H, CH, CH_2_), 1.49 – 1.20 (m, 54H, CH_2_), 1.03 – 0.85 (m, 18H, CH_3_).

**The general synthesis of compound G-D-CH_3_:**

Under the atmosphere of nitrogen, the mixture of **G-D-Br** (1.00 equiv.), (4-(methoxycarbonyl)phenyl)boronic acid (1.30 equiv.), K_2_CO_3_(4.00 equiv.), and Pd(amphos)Cl_2_ (0.10 equiv.) in deaerating THF: H_2_O = 10:1 (10 mL) was placed in a Schlenk tube and stirred at 80 ^o^C for 6 h. After being cooled to room-temperature, the mixture was poured into water and extracted with dichloromethane for three times. The organic phase was evaporated under vaccuum and purified by column chromatography on silica gel.

**G-TPA-CH_3_**: Red solid (55 mg, 92%). ^1^H NMR (400 MHz, CDCl_3_) δ (ppm): 8.39 (d, *J* = 5.1 Hz, 2H, ArH), 8.10 (d, *J* = 8.4 Hz, 2H, ArH), 7.62 (d, *J* = 8.4 Hz, 2H, ArH), 7.38 (d, *J* = 8.7 Hz, 2H, ArH), 7.32 – 7.26 (m, 4H, ArH), 7.13 (d, *J* = 7.5 Hz, 4H, ArH), 7.09 – 7.04 (m, 2H, ArH), 7.02 (d, *J* = 8.7 Hz, 2H, ArH), 4.15 (t, *J* = 6.9 Hz, 4H, OCH_2_), 3.95 (s, 3H, OCH_3_), 2.76 (m, 4H, CH_2_), 1.96 (d, *J* = 7.1 Hz, 4H, CH_2_), 1.74 (m, 2H, CH), 1.51 – 1.16 (m, 52H, CH_2_), 0.98 – 0.77 (m, 18H, CH_3_). ^13^C NMR (100 MHz, CDCl_3_) δ (ppm): 170.80, 152.05, 151.96, 150.90, 150.82, 147.88, 147.21, 146.24, 140.44, 139.17, 138.89, 134.25, 133.63, 133.30, 132.66, 132.23, 130.38, 129.42, 129.33, 125.00, 123.58, 122.37, 121.35, 116.34, 97.34, 82.44, 74.49, 40.65, 40.58, 33.96, 33.00, 32.74, 32.57, 31.96, 30.43, 29.76, 29.67, 29.40, 28.97, 28.65, 26.12, 26.04, 25.89, 25.80, 23.19, 23.09, 22.72, 14.22, 14.14, 10.99, 10.79. MS (MALDI-TOF, *m/z*): [M]^+^ calcd. for C_82_H_107_N_3_O_4_S_3_, 1293.742; found, 1293.685.

**G-DMAC-CH_3_**: Red solid (54 mg, 89%). ^1^H NMR (400 MHz, CDCl_3_) δ (ppm): 8.44 (d, *J* = 8.3 Hz, 2H, ArH), 8.12 (d, *J* = 8.4 Hz, 2H, ArH), 7.80 (d, *J* = 8.4 Hz, 2H, ArH), 7.63 (d, *J* = 8.4 Hz, 2H, ArH), 7.48 (d, *J* = 9.2 Hz, 2H, ArH), 7.36 (d, *J* = 8.4 Hz, 2H, ArH), 7.04 – 6.92 (m, 4H, ArH), 6.33 (d, *J* = 9.4 Hz, 2H, ArH), 4.18 (q, *J* = 6.9 Hz, 4H, OCH_2_), 3.97 (s, 3H, OCH_3_), 2.81 (m, 4H, CH_2_), 1.99 (m, 4H, CH_2_), 1.86 (s, 1H, CH), 1.71 (s, 6H, CH_3_), 1.68 (s, 1H, CH), 1.52 – 1.21 (m, 52H, CH_2_), 1.01 – 0.82 (m, 18H, CH_3_). ^13^C NMR (100 MHz, CDCl_3_) δ (ppm): 166.94, 152.26, 151.87, 150.90, 150.79, 147.18, 141.03, 140.71, 139.72, 139.39, 138.77, 134.55, 134.30, 133.72, 133.07, 132.63, 131.52, 130.12, 129.81, 129.29, 128.78, 126.43, 125.31, 123.54, 120.76, 120.42, 117.64, 117.07, 114.05, 96.21, 84.51, 74.52, 52.20, 40.76, 40.56, 36.01, 34.05, 32.95, 32.78, 32.56, 31.96, 31.25, 30.43, 29.76, 29.71, 29.66, 29.41, 29.03, 28.64, 26.12, 26.06, 25.94, 25.78, 23.21, 23.09, 22.84, 22.72, 14.25, 14.15, 11.04, 10.78. MS (MALDI-TOF, *m/z*): [M]^+^ calcd. for C_85_H_111_N_3_O_4_S_3_, 1334.777; found, 1334.884.

**G-PTZ-CH_3_**: Red solid (54 mg, 90%). ^1^H NMR (400 MHz, CDCl_3_) δ (ppm): 8.41 (d, *J* = 3.9 Hz, 2H, ArH), 8.11 (d, *J* = 8.3 Hz, 2H, ArH), 7.71 (d, *J* = 8.4 Hz, 2H, ArH), 7.62 (d, *J* = 8.3 Hz, 2H, ArH), 7.35 (d, *J* = 8.4 Hz, 2H, ArH), 7.13 – 7.05 (m, 2H, ArH), 6.99 – 6.83 (m, 4H, ArH), 6.39 (d, *J* = 9.2 Hz, 2H, ArH), 4.17 (q, *J* = 7.0 Hz, 4H, OCH_2_), 3.96 (s, 3H, OCH_3_), 2.78 (m, 4H, CH_2_), 2.03 – 1.91 (m, 4H, CH_2_), 1.83 (s, 1H, CH), 1.68 (s, 1H, CH), 1.49 – 1.17 (m, 52H, CH_2_), 1.01 – 0.77 (m, 18H, CH_3_). ^13^C NMR (100 MHz, CDCl_3_) δ (ppm): 166.94, 152.26, 151.87, 150.90, 150.78, 147.09, 143.74, 141.35, 139.72, 139.39, 138.77, 134.48, 134.29, 133.42, 133.07, 132.61, 129.80, 129.28, 129.15, 128.78, 127.08, 126.95, 123.08, 122.60, 122.05, 120.46, 117.63, 117.39, 117.07, 96.20, 84.36, 74.46, 52.19, 40.73, 40.56, 34.03, 32.96, 32.76, 32.56, 31.95, 30.42, 29.75, 29.70, 29.65, 29.40, 29.01, 28.64, 26.11, 26.04, 25.92, 25.78, 23.19, 23.08, 22.71, 14.23, 14.14, 11.01, 10.77. MS (MALDI-TOF, *m/z*): [M]^+^ calcd. for C_82_H_105_N_3_O_4_S_4_, 1324.702; found, 1324.650.

**G-CS-CH_3_**: Red solid (56 mg, 88%). ^1^H NMR (400 MHz, CDCl_3_) δ (ppm): 8.43 (d, *J* = 9.1 Hz, 2H, ArH), 8.19 (dd, *J* = 7.9, 1.4 Hz, 2H, ArH), 8.11 (d, *J* = 8.3 Hz, 2H, ArH), 7.84 (d, *J* = 8.4 Hz, 2H, ArH), 7.62 (d, *J* = 8.3 Hz, 2H, ArH), 7.45 – 7.38 (m, 4H, ArH), 7.28 (d, *J* = 7.7 Hz, 2H, ArH), 6.68 (d, *J* = 8.7 Hz, 2H, ArH), 4.18 (q, *J* = 7.2 Hz, 4H, OCH_2_), 3.96 (s, 3H, OCH_3_), 2.80 (m, 4H, CH_2_), 1.98 (m, 4H, CH_2_), 1.85 (s, 1H, CH), 1.68 (s, 1H, CH), 1.50 – 1.23 (m, 52H, CH_2_), 1.00 – 0.79 (m, 18H, CH_3_). ^13^C NMR (100 MHz, CDCl_3_) δ (ppm): 166.92, 152.40, 151.81, 150.89, 150.75, 147.67, 140.60, 139.67, 139.50, 138.79, 138.25, 135.10, 134.36, 133.96, 132.98, 132.86, 132.60, 130.72, 129.80, 129.28, 128.81, 125.40, 123.55, 122.77, 122.25, 119.83, 117.83, 117.12, 116.89, 95.39, 85.79, 74.57, 74.54, 52.20, 40.78, 40.54, 34.09, 32.95, 32.77, 32.55, 31.94, 30.42, 29.74, 29.69, 29.64, 29.39, 29.03, 28.63, 26.11, 26.04, 25.93, 25.77, 23.19, 23.07, 22.70, 14.24, 14.13, 11.03, 10.77. MS (MALDI-TOF, *m/z*): [M]^+^ calcd. for C_82_H_105_N_3_O_6_S_4_, 1355.689; found, 1355.663.

**The general synthesis of compounds G-D:**

The mixture of **G-D-CH_3_** (1.00 equiv.), KOH (10.00 equiv.), THF (6 mL) and H_2_O (6 mL) was placed in a round-bottom flask and stirred at 80 ^o^C for 24 h. After being cooled to room-temperature, the mixture was poured into diluted hydrochloric acid, and extracted by dichloromethane for three times. The organic phase was evaporated under vacuum and purified by column chromatography on silica gel.

**G-TPA**: Red solid (118 mg, 87%). ^1^H NMR (400 MHz, CDCl_3_) δ (ppm): 8.41 (m, 2H, ArH), 8.20 (d, *J* = 8.2 Hz, 2H, ArH), 7.68 (d, *J* = 8.3 Hz, 2H, ArH), 7.39 (d, *J* = 8.7 Hz, 2H, ArH), 7.29 (dd, *J* = 8.4, 7.4 Hz, 4H, ArH), 7.13 (d, *J* = 7.5 Hz, 4H, ArH), 7.10 – 7.00 (m, 4H, ArH), 4.17 (q, *J* = 6.8 Hz, 4H, OCH_2_), 2.78 (t, *J* = 7.2 Hz, 4H, CH_2_), 2.06 – 1.90 (m, 4H, CH_2_), 1.76 (m, 2H, CH), 1.52 – 1.23 (m, 52H, CH_2_), 0.98 – 0.80 (m, 18H, CH_3_). ^13^C NMR (100 MHz, CDCl_3_) δ (ppm): 152.04, 151.98, 150.89, 150.82, 147.88, 147.21, 146.24, 140.66, 139.09, 138.95, 134.25, 133.62, 133.37, 132.66, 132.23, 130.45, 129.42, 129.36, 125.00, 123.57, 122.37, 121.36, 117.33, 116.34, 97.34, 82.43, 74.50, 40.65, 40.58, 33.96, 33.01, 32.73, 32.57, 31.96, 30.43, 30.41, 29.75, 29.73, 29.64, 29.40, 28.96, 28.65, 26.12, 26.03, 25.88, 25.80, 23.18, 23.09, 22.72, 14.22, 14.14, 10.98, 10.79. MS (MALDI-TOF, *m/z*): [M]^+^ calcd. for C_81_H_105_N_3_O_4_S_3_, 1280.730; found, 1280.617.

**G-DMAC**: Red solid (38 mg, 88%). ^1^H NMR (400 MHz, CDCl_3_) δ (ppm): 8.42 (d, *J* = 6.2 Hz, 2H, ArH), 8.18 (d, *J* = 7.9 Hz, 2H, ArH), 7.79 (d, *J* = 8.2 Hz, 2H, ArH), 7.65 (d, *J* = 8.1 Hz, 2H, ArH), 7.47 (d, *J* = 8.5 Hz, 2H, ArH), 7.35 (d, *J* = 8.2 Hz, 2H, ArH), 6.97 (m, 4H, ArH), 6.32 (d, *J* = 8.1 Hz, 2H, ArH), 4.18 (q, *J* = 6.6 Hz, 4H, OCH_2_), 2.85 (d, *J* = 4.5 Hz, 2H, CH_2_), 2.76 (d, *J* = 7.0 Hz, 2H, CH_2_), 1.98 (m, 4H, CH_2_), 1.85 (s, 1H, CH), 1.70 (s, 6H, CH_3_), 1.67 (s, 1H, CH), 1.48 – 1.24 (m, 52H, CH_2_), 1.00 (t, *J* = 7.4 Hz, 3H, CH_3_), 0.85 (m, 15H, CH_3_). ^13^C NMR (100 MHz, CDCl_3_) δ (ppm): 152.27, 151.87, 150.90, 150.78, 147.17, 141.03, 140.70, 134.55, 134.28, 133.70, 133.10, 132.56, 131.51, 130.32, 130.12, 129.28, 126.42, 125.30, 120.75, 120.41, 117.65, 117.34, 117.05, 114.04, 74.50, 40.75, 40.57, 36.00, 34.05, 32.98, 32.77, 32.56, 31.95, 31.24, 30.42, 29.70, 29.65, 29.39, 29.02, 28.65, 26.11, 26.05, 25.93, 25.79, 23.20, 23.08, 22.70, 14.23, 14.14, 11.03, 10.79. MS (MALDI-TOF, *m/z*): [M]^+^ calcd. for C_84_H_109_N_3_O_4_S_3_, 1319.758; found, 1319.719.

**G-PTZ**: Red solid (35 mg, 92%). ^1^H NMR (400 MHz, CDCl_3_) δ (ppm): 8.43 (d, *J* = 2.3 Hz, 2H, ArH), 8.19 (d, *J* = 8.2 Hz, 2H, ArH), 7.69 (m, 4H, ArH), 7.35 (d, *J* = 8.3 Hz, 2H, ArH), 7.08 (d, *J* = 8.5 Hz, 2H, ArH), 6.90 (m, 6.9 Hz, 4H, ArH), 6.39 (d, *J* = 8.0 Hz, 2H, ArH), 4.18 (t, *J* = 6.8 Hz, 4H, OCH_2_), 2.80 (m, 4H, CH_2_), 2.03 – 1.94 (m, 4H, CH_2_), 1.83 (s, 1H, CH), 1.70 (s, 1H, CH), 1.49 – 1.21 (m, 52H, CH_2_), 0.98 (t, *J* = 7.4 Hz, 3H CH_3_), 0.85 (m, 15H, CH_3_). ^13^C NMR (100 MHz, CDCl_3_) δ (ppm): 171.15, 152.24, 151.92, 150.89, 150.79, 147.10, 143.74, 141.36, 140.62, 139.22, 138.98, 134.47, 134.35, 133.43, 133.29, 132.64, 130.45, 129.36, 129.15, 127.84, 127.08, 126.96, 123.09, 122.59, 122.06, 120.50, 117.58, 117.40, 117.14, 96.22, 84.35, 74.54, 40.73, 40.59, 34.04, 33.00, 32.76, 32.57, 31.95, 30.42, 29.73, 29.65, 29.01, 28.66, 26.13, 26.05, 25.80, 23.19, 23.09, 22.71, 14.23, 14.14, 11.01, 10.80. MS (MALDI-TOF, *m/z*): [M]^+^ calcd. for C_81_H_103_N_3_O_4_S_4_, 1310.686; found, 1310.554.

**G-CS**: Red solid (102 mg, 94%). ^1^H NMR (400 MHz, CDCl_3_) δ (ppm): 8.44 (d, *J* = 3.1 Hz, 2H, ArH), 8.19 (d, *J* = 8.9 Hz, 4H, ArH), 7.85 (d, *J* = 8.3 Hz, 2H, ArH), 7.68 (d, *J* = 7.9 Hz, 2H, ArH), 7.48 – 7.38 (m, 4H, ArH), 7.29 (d, *J* = 7.5 Hz, 2H, ArH), 6.69 (d, *J* = 8.7 Hz, 2H, ArH), 4.19 (q, *J* = 6.9 Hz, 4H, OCH_2_), 2.85 (d, *J* = 6.3 Hz, 2H, CH_2_), 2.77 (d, *J* = 6.9 Hz, 2H, CH_2_), 2.04 – 1.94 (m, 4H, CH_2_), 1.85 (s, 1H, CH), 1.69 (d, *J* = 6.0 Hz, 1H, CH), 1.48 – 1.21 (m, 52H, CH_2_), 1.00 (t, *J* = 7.4 Hz, 3H, CH_3_), 0.92 – 0.79 (m, 15H, CH_3_). ^13^C NMR (100 MHz, CDCl_3_) δ (ppm): 152.39, 151.86, 150.88, 150.75, 147.68, 140.60, 139.31, 138.99, 138.49, 138.26, 135.76, 135.08, 134.41, 133.96, 133.38, 133.21, 132.87, 132.62, 130.73, 130.44, 129.37, 127.76, 125.40, 123.56, 122.77, 122.26, 117.12, 116.96, 74.56, 40.78, 34.09, 32.99, 32.77, 32.57, 31.94, 30.42, 29.75, 29.70, 29.65, 29.40, 29.03, 28.65, 26.12, 26.04, 25.94, 25.79, 23.19, 23.08, 22.70, 14.24, 14.13, 11.03, 10.79. MS (MALDI-TOF, *m/z*): [M]^+^ calcd. for C_81_H_103_N_3_O_6_S_4_, 1341.673; found, 1341.665.

**Statement of ethical approval**

All animal studies were performed according to the guidelines, and the overall project protocols were approved by the Welfare and Ethics Committee of Laboratory Animal, College of Life Sciences, Wuhan University (approval no. WDSKY0202101).

**Supplemental Figures**

**Figure S1.** The dihedral angles of involved phenyl (blue plane), and 9,9-dimethylacridan, phenothiazine, phenothiazine 5,5-dioxide or diphenylamine (yellow plane) of G-D-CH_3_ series and G-D series.

**Table S1**. Photophysical properties of luminogen G-D-CH_3_ and G-D

|  | Solution | | | Solid | | | | | |
| --- | --- | --- | --- | --- | --- | --- | --- | --- | --- |
| Compound | 298 K | 77 K | | 298 K | | | | | |
|  | *^a^λ*_F_  (nm) | *^b^λ*_F_  (nm) | *^b^λ*_P_  (nm) | *^c^λ*_F_  (nm) | *^c^λ*_P_  (nm) | *^c^ τ*_P1_ (ms) | *^c^τ*_P2_  (μs) | *^c^* PLQY  (%) | |
| G-TPA-CH_3_ | 625 | 595 | 515, 549 | 652 | 658, 740 | 54 | 3.32 | | 30% |
| G-TPA | 624 | 595 | 510, 560 | 665 | 667, 740 | 31 | 2.86 | | 51% |
| G-DMAC-CH_3_ | 594 | 579 | 505, 556 | 625 | 645, 730 | 64 | 7.70 | | 45% |
| G-DMAC | 594 | 571 | 500, 550 | 632 | 642, 720 | 39 | 7.84 | | 48% |
| G-PTZ-CH_3_ | 613 | 587 | 495, 551 | 636 | 645, 720 | 52 | 8.39 | | 28% |
| G-PTZ | 608 | 583 | 498, 521 | 645 | 647, 710 | 43 | 4.33 | | 19% |
| G-CS-CH_3_ | 586 | 573 | 508, 542, 689 | 630 | 636, 713 | 118 | 17.92 | | 33% |
| G-CS | 587 | 574 | 508, 548, 690 | 632 | 650, 720 | 46 | 10.29 | | 25% |

*^a^*In THF solution with concentration of 1×10^−5^ mol L^−1^ at room temperature. *^b^*In THF solution with concentration of 1×10^−5^ mol L^−1^ at 77 K. *^c^* Solid state at room temperature*. λ*_F_: The emission wavelength of fluorescence spectra. *λ*_P_: The emission wavelength of phosphorescence spectra. *τ*_P1_: RTP lifetime at 636-667 nm. *τ*_P2_: RTP lifetime at 710-740 nm. PLQY: Photoluminescence quantum yield.


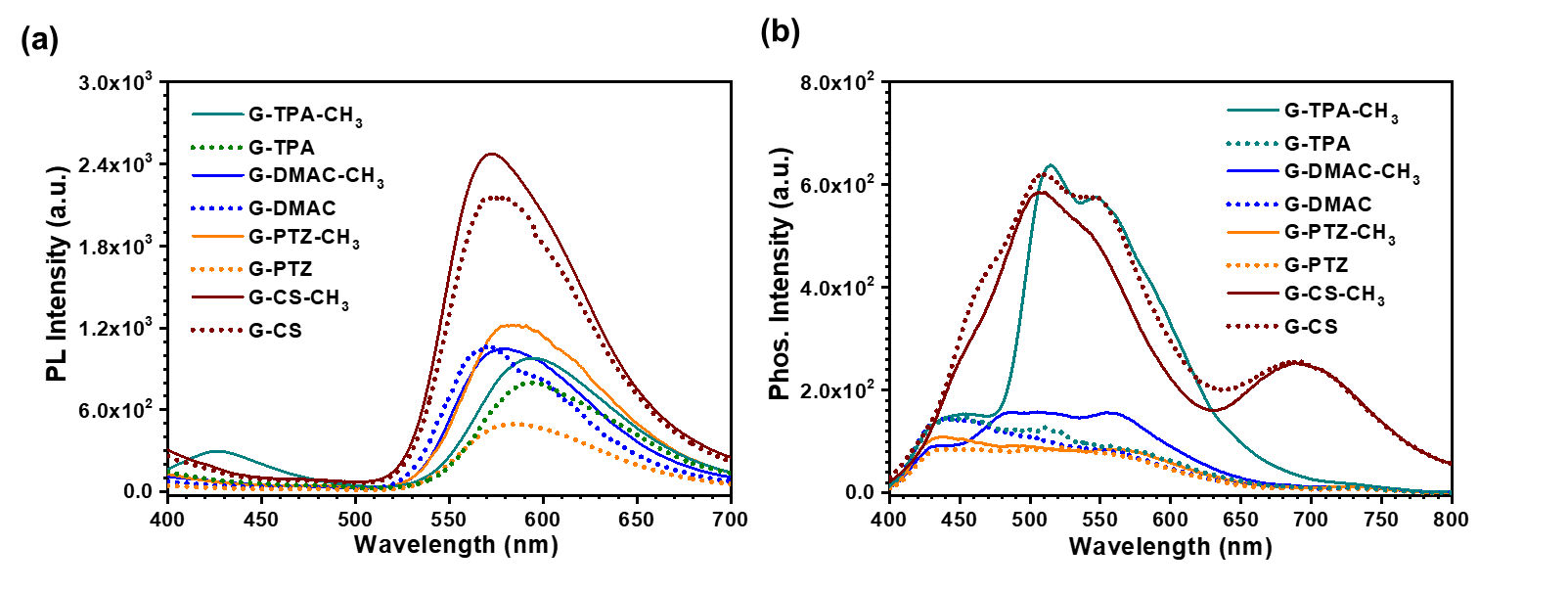


**Figure S2**. (a) PL spectra of luminogen G-D-CH_3_ and G-D in THF solution with concentration of 1×10^−5^ mol L^−1^ at 77 K. (b) Phosphorescence spectra of luminogen G-D-CH_3_ and G-D in THF solution with concentration of 1×10^−5^ mol L^−1^ at 77 K.

**Figure S3**. Photographs of luminogen G-D-CH_3_ and G-D under UV light and day light, and afterglow photographs at different times after ceasing white light, captured by a chemiluminescence imager.

**Figure S4**. PLQYs of luminogen G-D-CH_3_ and G-D at solid state.


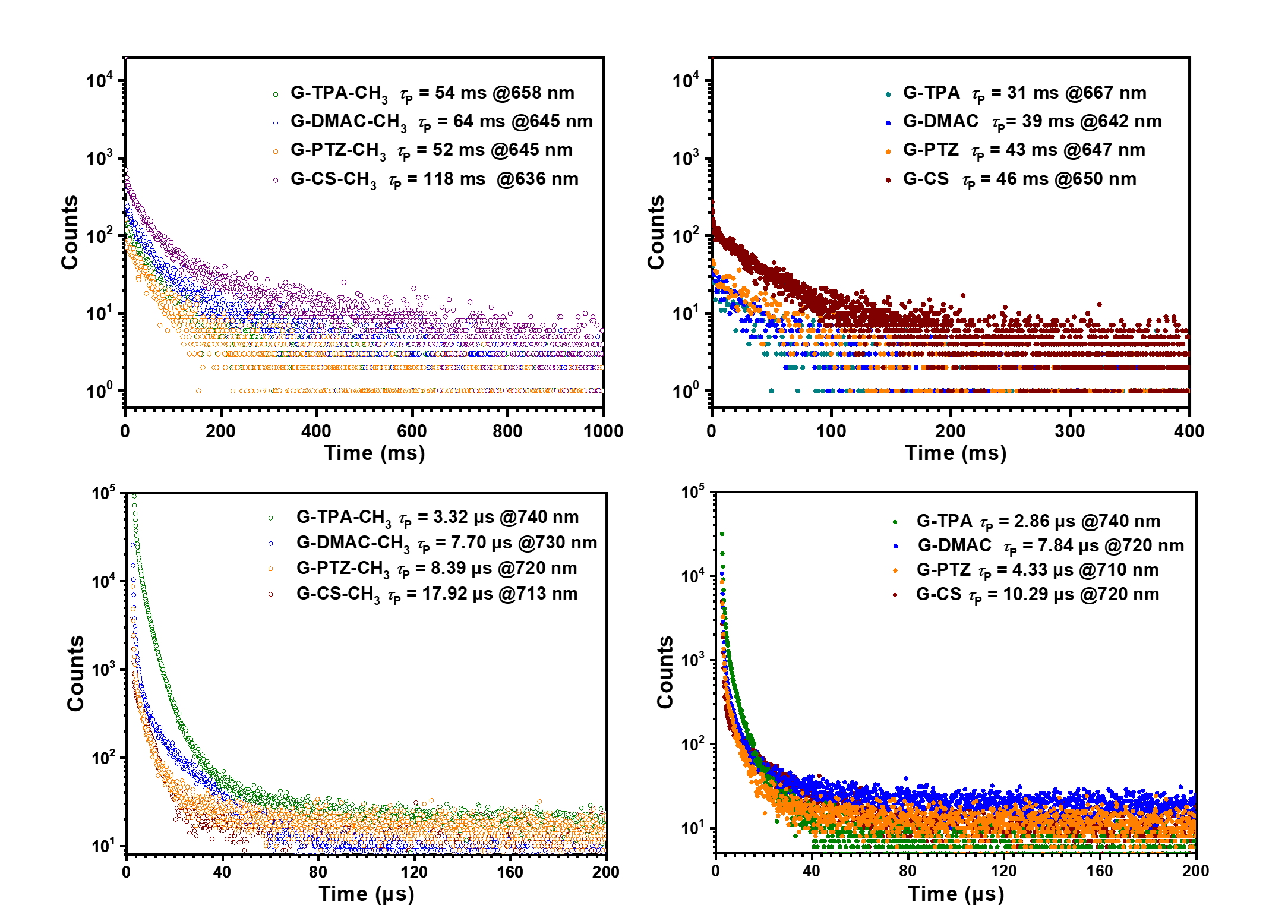


**Figure S5**. Time-dependent phosphorescence decays of luminogen G-D-CH_3_ and G-D.


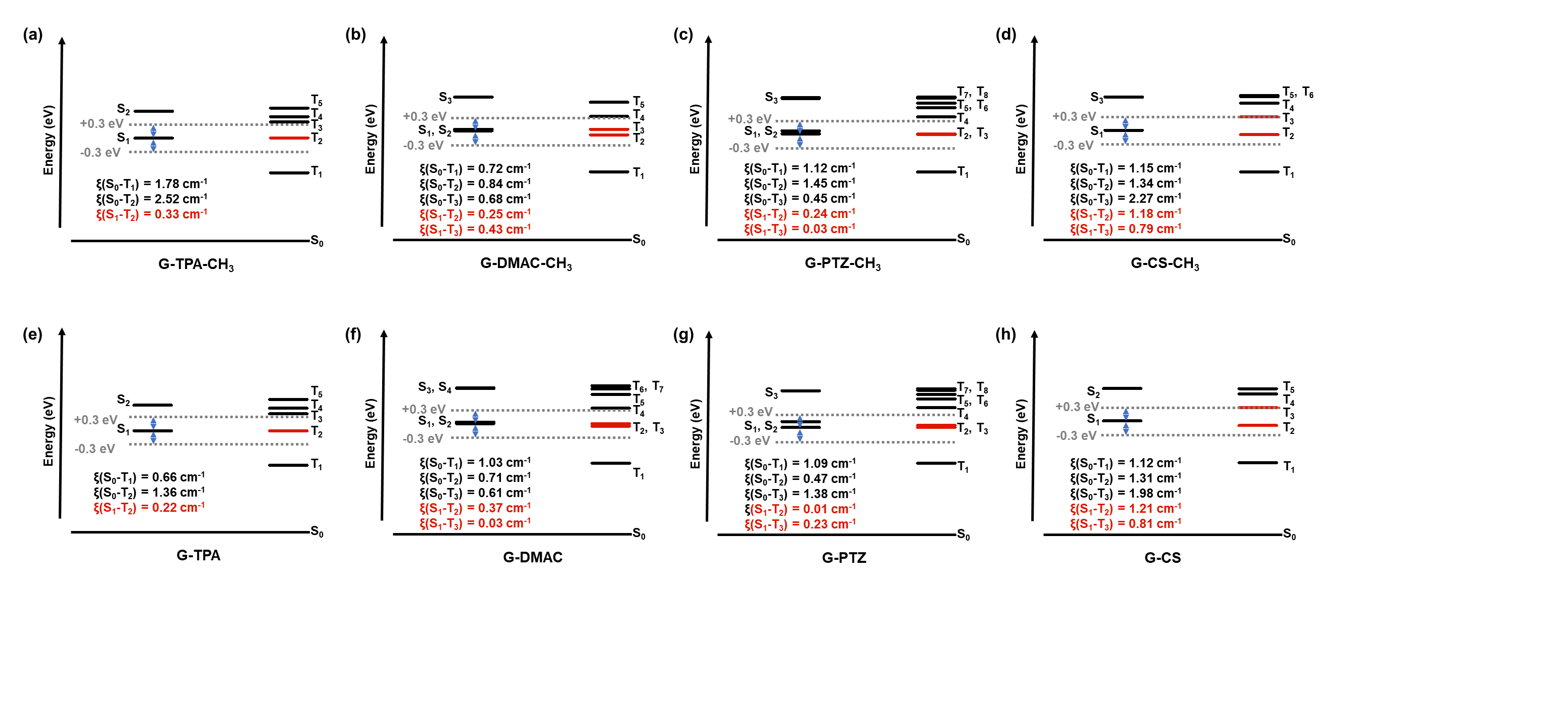


**Figure S6**. Calculated energy levels and corresponding SOC constants (ξ) of (a) G-TPA-CH_3_, (b) G-DMAC-CH_3_, (c) G-PTZ-CH_3_, (d) G-CS-CH_3_, (e) G-TPA, (f) G-DMAC, (g) G-PTA and (h) G-CS in isolate state using the PBE0 functional with def2svp basis. T_n_ relevant to possible ISC channels and the corresponding SOC were marked by red color.


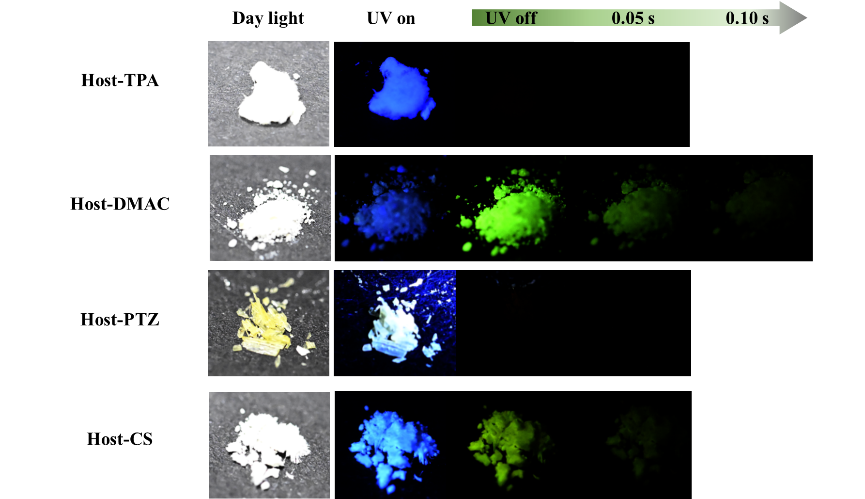


**Figure S7.** Photographs of Host-D under day light and UV light, and afterglow photographs at different times after ceasing UV light, captured by a camera.

**Figure S8**. (a) Time-dependent fluorescence decays of Host-D at solid states. (b) Time-dependent phosphorescence decays of Host-D at solid state.

**Figure S9**. (a) UV–Vis absorption spectra of Host-D in THF solution with concentration of 1×10^−5^ mol L^−1^. (b) PL spectra of Host-D in THF solution with concentration of 1×10^−5^ mol L^−1^ at room temperature. (c) PL spectra of Host-D in THF solution with concentration of 1×10^−5^ mol L^−1^ at 77 K. (d) Phosphorescence spectra of Host-D in THF solution with concentration of 1×10^−5^ mol L^−1^ at 77 K.

**Figure S10**. Molecular conformations, packing modes and intermolecular interactions of Host-D in the single crystals. (The dihedral angles of two phenyl planes of 9,9-dimethylacridan, phenothiazine, phenothiazine 5,5-dioxide and diphenylamine were measured by Murcury 2022.2.0. The torsions of 9,9-dimethylacridan, phenothiazine, phenothiazine 5,5-dioxide, diphenylamine and involved phenyl were marked with red circle and measured by Murcury 2022.2.0).

**Figure S11**. (a) PL spectra of Host-D at solid states at 77 K. (b) Phosphorescence spectra of Host-D at the solid state at 77 K (Insert: Partial amplifie view of Phosphorescence spectra). (c–i) Time-dependent phosphorescence decays of Host-D at the solid state at 77 K.

**Table S2**. Photophysical properties of Host-D.

| Compound | Solid (298 K) | | | | | Solution (77K) | Solid (77 K) | |
| --- | --- | --- | --- | --- | --- | --- | --- | --- |
|  | *λ*_F_ (nm) | *λ*_f_ (ns) | *λ*_P_ (nm) | *τ*_P_ (ms) | PLQY (%) | *λ*_P_ (nm) | *λ_P_* (nm) | *τ*_P_ (ms) |
| Host-TPA | 420 | 0.60 | 560 | 2.21 | 8.81% | 459, 491 | 505 | 516 |
| Host-DMAC | 410 | 14.90 | 550 | 5.36 | 3.21% | 393, 450 | 470 | 572 |
| Host-PTZ | 480 | 3.90 | 600 | 6.20 | 0.6% | 435, 497 | 511, 547 | 44, 182 |
| Host-CS | 398 | 1.30 | 536 | 1.69 | 2.04% | 415, 440 | 440, 486 | 1560, 520 |

*λ*_F_: The emission wavelength of fluorescence spectra. *τ*_f_: fluorescence lifetime. *λ*_P_: The emission wavelength of phosphorescence spectra. *τ*_P_: RTP lifetime. PLQY: Photoluminescence quantum yield.

**Figure S12.** (a) Normalized PL spectra of Host-D at solid state and the absorption spectrum of G-CS as an example of guests, which demonstrated the similar absorption spectra. (b) Normalized phosphorescence spectra of Host-D at solid state and the absorption spectrum of G-CS as an example of guests, which demonstrated the similar absorption spectra.

**Figure S13**. Afterglow photographs of Host-CS/G-CS with different mass ratios at different times after ceasing white-light, captured by a chemiluminescence imager.

**Figure S14**. (a) PL spectra of Host-CS/G-CS with mass ratio of 1:1, 10:1 and 100:1. (b) Phosphorescence spectra of Host-CS/G-CS with mass ratio of 1:1, 10:1 and 100:1. (c) Time-dependent phosphorescence decays of Host-CS/G-CS with mass ratio of 1:1, 10:1 and 100:1 at 630 nm-650 nm. (d) Time-dependent phosphorescence decays of Host-CS/G-CS with mass ratio of 10:1 and 100:1 at about 750 nm (Host-CS/G-CS with mass ratio of 1:1 with no emission peaks at about 750 nm).

**Figure S15**. (a) PL spectra of Host-D/G-CS-CH_3_. (b) PL spectra of Host-D/G-CS. (c) Time-dependent phosphorescence decays of Host-D/G-CS-CH_3_ at 615 nm-645 nm. (d) Time-dependent phosphorescence decays of Host-D/G-CS at 650 nm. (e) Time-dependent phosphorescence decays of Host-D/G-CS-CH_3_ at 690 nm-763 nm. (f) Time-dependent phosphorescence decays of Host-D/G-CS at 750 nm.

**Table S3**. Phosphorescence resonance energy transfer in host-guest systems

| Host (Donor) | Guest (Acceptor) | *λ*_P_ | *τ*_D_ | *τ*_DA_ | *Φ*_ET_ |
| --- | --- | --- | --- | --- | --- |
| Host-TPA | G-CS-CH_3_ | 560 nm | 2.21 ms | 10 μs | 99.5% |
| Host-DMAC |  | 550 nm | 5.36 ms | 12 μs | 99.8% |
| Host-PTZ |  | 600 nm | 6.20 ms | 24 μs | 99.6% |
| Host-CS |  | 536 nm | 1.69 ms | 13 μs | 99.2% |
| Host-TPA | G-CS | 560 nm | 2.21 ms | 11 μs | 99.5% |
| Host-DMAC |  | 550 nm | 5.36 ms | 12 μs | 99.7% |
| Host-PTZ |  | 600 nm | 6.20 ms | 11 μs | 99.8% |
| Host-CS |  | 536 nm | 1.69 ms | 14 μs | 99.2% |

*λ*_P_: the emission wavelength of phosphorescence spectra of hosts. *τ*_D:_ RTP lifetime of hosts at *λ*_P._ *τ*_DA_: RTP lifetime of host-guest systems at *λ*_P._ *Φ*_ET_: Efficiency of energy transfer between host as energy donor and guest as energy acceptor, calculated from the equation: *Φ*_ET_ = 1-*τ*_DA_/*τ*_D._


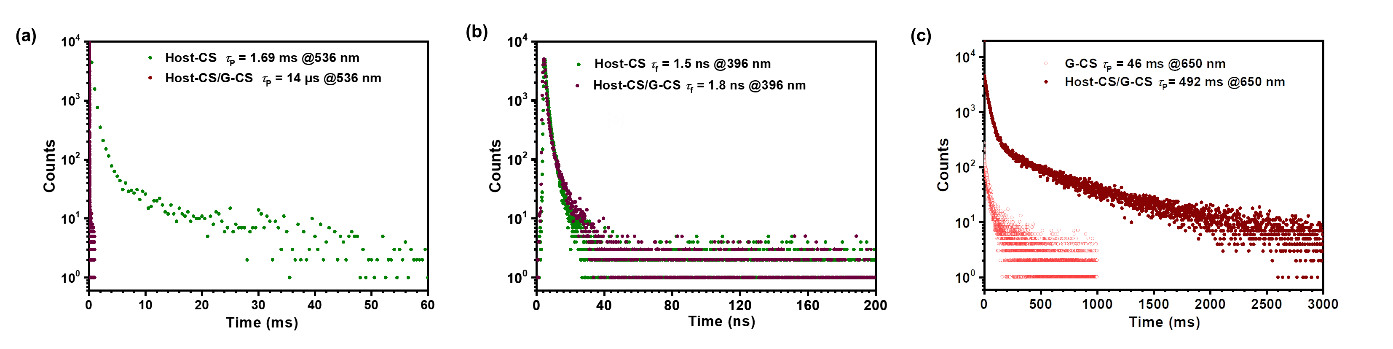


**Figure S16**. (a) Time-dependent decays of Host-CS and Host-CS/G-CS at 536 nm at solid states. (b) Time-dependent decays of Host-CS and Host-CS/G-CS at 396 nm at solid states. (c) Time-dependent decays of G-CS and Host-CS/G-CS at 650 nm at solid states.

**Figure S17**. The calculated energy levels and possible energy transfer processes in Host-CS/G-CS. (TSET and TTET refer to the energy transfer process of triplet-to-singlet and triplet-to-triplet excited states, respectively)


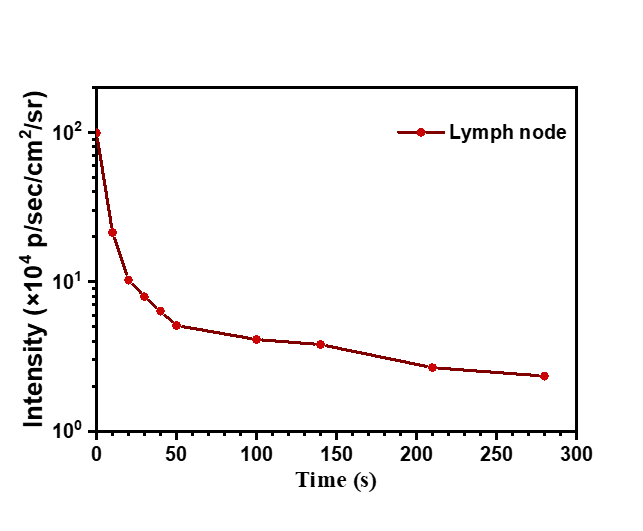


**Figure S18**. Time-dependent afterglow decay of *in vivo* imaging of lymph node in living mice by Host-CS/G-CS nanoparticles.

**Figure S19**. (a) PXRD pattern of Host-TPA (the simulated PXRD pattern calculated from single-crystal X-ray data with Mercury 2022.2.0) and Host-TPA/G-CS. (b) PXRD pattern of Host-DMAC (the simulated PXRD pattern calculated from single-crystal X-ray data with Mercury 2022.2.0) and Host-DMAC/G-CS. (c) PXRD pattern of Host-PTZ (the simulated PXRD pattern calculated from single-crystal X-ray data with Mercury 2022.2.0) and Host-PTZ/G-CS. (d) PXRD pattern of Host-CS (the simulated PXRD pattern calculated from single-crystal X-ray data with Mercury 2022.2.0) and Host-CS/G-CS (Violet background refers to the diffraction peaks with obvious changes).

**Table S4.** Data of Host-D and Ref-Host-CS crystals

| Name | Host-TPA | Host-DMAC | Host-PTZ | Host-CS | Ref-Host-CS |
| --- | --- | --- | --- | --- | --- |
| Formula | C_20_H_15_N | C_23_H_19_N | C_20_H_13_NS | 2(C_20_H_13_NO_2_S), CH_2_Cl_2_ | 2(C_19_H_15_NO_2_S) |
| Wavelength(Å) | 1.54184 | 1.54184 | 1.54184 | 1.54184 | 1.54184 |
| Space Group | P c a 21 | P 21/c | P 21/n | P -1 | P 21/n |
| Cell Lengths(Å) | a = 9.1339 (3)  b = 17.1566 (5)  c = 19.4244 (9) | a = 9.5196 (4)  b = 22.8502 (10)  c = 8.4273 (4) | a = 7.18221 (7)  b = 29.7368 (3)  c = 7.26357 (7) | a = 11.6425 (5)  b = 13.2604 (6)  c = 13.3734 (7) | a = 16.8938 (2)  b = 10.3969 (14)  c = 18.1046 (7) |
| Cell Angles (^o^) | α = 90  β = 90  γ = 90 | α = 90  β = 107.521 (5)  γ = 90 | α = 90  β = 100.6999 (10)  γ = 90 | α = 115.098 (5)  β = 94.195 (4)  γ = 104.656 (4) | α = 90  β = 104.5577 (17)  γ = 90 |
| Cell Volume(Å^3^) | 3043.93 (19) | 1748.10 (14) | 1524.35 (3) | 1770.08 (18) | 3078.11 (7) |
| Z | 8 | 4 | 4 | 2 | 4 |
| Density (g/cm^3^) | 1.175 | 1.176 | 1.304 | 1.403  _(calculated for 1.126 by removing the CH2Cl2)_ | 1.387 |
| F (000) | 1136.0 | 656.0 | 624.0 | 772.0 | 1344.0 |
| *h*_max_, *k*_max_, *I*_max_ | 10, 20, 23 | 11, 28, 10 | 8, 35, 8 | 13, 15, 15 | 20, 12, 21 |
| CCDC Number | 2334438 | 2334436 | 2334439 | 2334437 | 1519853 |

**Figure S20**. (a) Phosphorescence spectra of G-CS at 30 ^o^C and after thermal annealing at 230 ^o^C. (b) Phosphorescence spectra of Host-CS/G-CS at 30 ^o^C and after thermal annealing at 230 ^o^C. (c) Phosphorescence lifetimes of G-CS and Host-CS/G-CS at 650 nm at 30 ^o^C and after thermal annealing at 230 ^o^C.


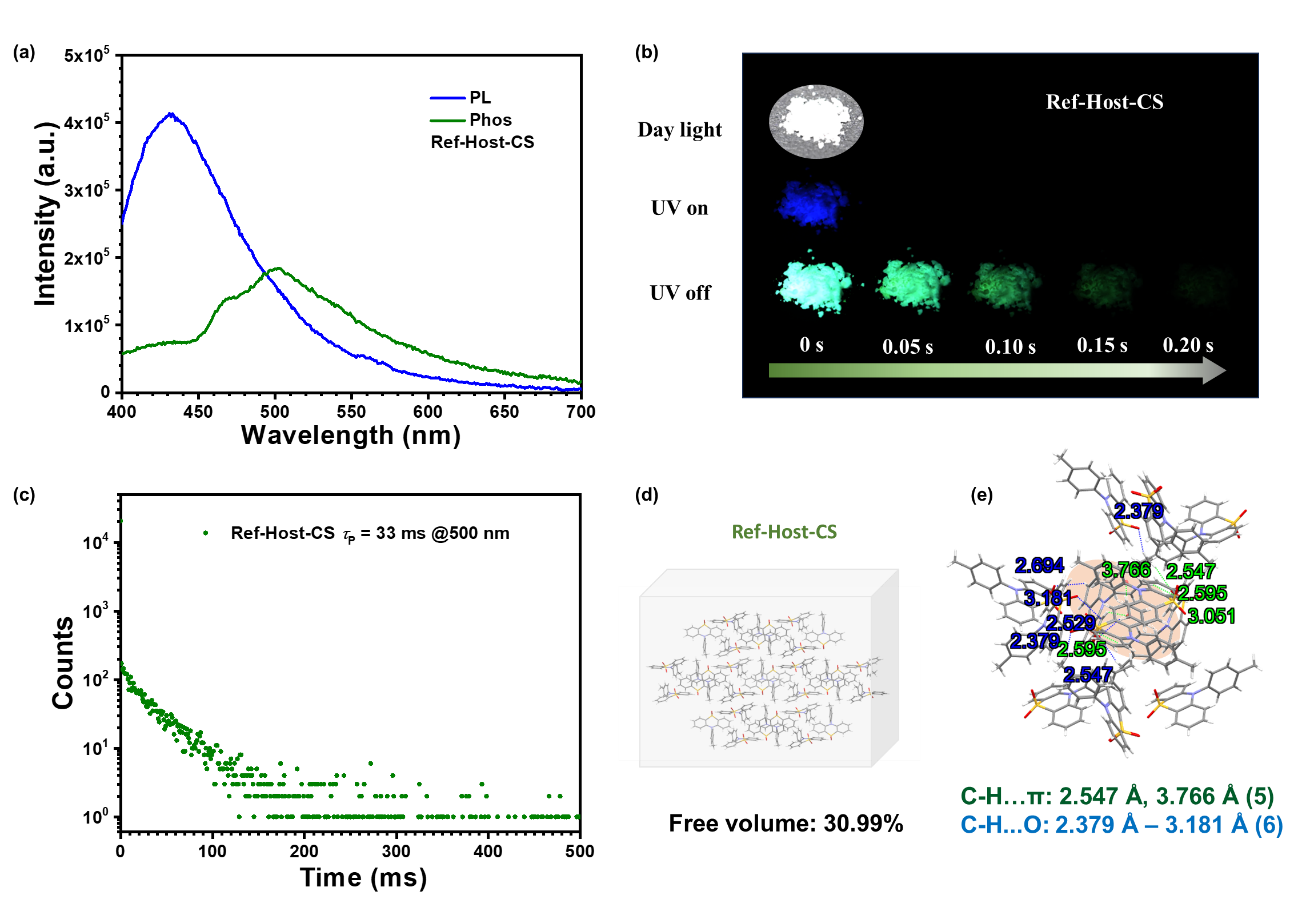


**Figure S21**. (a) PL and phosphorescence spectra of Ref-Host-CS at solid state. (b) Photographs of Ref-Host-CS under day light and UV light, and afterglow photographs at different times after ceasing UV light, captured by a camera. (c) Time-dependent phosphorescence decay of Ref-Host-CS at solid state. (d) Molecular packing mode and fractional free volume of Ref-Host-CS in crystal state. (e) Intermolecular interactions of central molecule with adjacent molecules in crystal state.

**Figure S22**. (a) PL and phosphorescence spectra of Ref-G-CS at solid state. (b) Time-dependent phosphorescence decay of Ref-G-CS at solid state at 630 nm. (c) Time-dependent phosphorescence decay of Ref-G-CS at solid state at 717 nm.

**Figure S23**. (a) Molecular structures of G-CS and Ref-G-CS. (b) The dihedral angles between the adjacent aromatic moieties in G-CS and Ref-G-CS (Numbers 1-6 refer to the different aromatics). (c) The dipole moments of G-CS and Ref-G-CS. (d) Absorption spectra of G-CS and Ref-G-CS in THF solution with the concentration of 1 × 10^−5^ mol L^−1^.

**Figure S24**. (a) PXRD pattern of Ref-Host-CS (the simulated PXRD pattern calculated from single-crystal X-ray data with Mercury 2022.2.0) and Ref-Host-CS/Ref-G-CS. (b) PXRD pattern of Ref-Host-CS (the simulated PXRD pattern calculated from single-crystal X-ray data with Mercury 2022.2.0) and Ref-Host-CS/G-CS. (c) PXRD pattern of Host-CS (the simulated PXRD pattern calculated from single-crystal X-ray data with Mercury 2022.2.0) and Host-CS/Ref-G-CS. (d) PXRD pattern of Host-CS (the simulated PXRD pattern calculated from single-crystal X-ray data with Mercury 2022.2.0) and Host-CS/G-CS (Violet background refers to the diffraction peaks with obvious changes).

**Figure S25**. (a) Molecular packing of Host-CS in single crystal and interspace. (b) Molecular packing of Ref-Host-CS in single crystal and interspace. The blue plane was described by the edge of benzene rings in each layer.

**Figure S26**. (a) PL spectra of Ref-Host-CS/G-CS in Air and under vacuum (Vac). (b) Phosphorescence spectra of Ref-Host-CS/G-CS in Air and under vacuum (Vac). (c) Time-dependent phosphorescence decay of Ref-Host-CS/G-CS in Air and under vacuum (Vac) at 650 nm. (d) PL spectra of Host-CS/G-CS in Air and under vacuum (Vac). (e) Phosphorescence spectra of Host-CS/G-CS in Air and under vacuum (Vac). (f) Time-dependent phosphorescence decay of Host-CS/G-CS in Air and under vacuum (Vac) at 650 nm.

**Figure S27.** Enhancement fold of RTP lifetimes from the atmosphere of air to vacuum or N_2_ in Ref-Host-CS/G-CS and Host-CS/G-CS.

**Figure S28**. (a) PL spectra of Host-CS/G-TPA, Host-CS/G-DMAC and Host-CS/G-PTZ. (b) Phosphorescence spectra of Host-CS/G-TPA, Host-CS/G-DMAC and Host-CS/G-PTZ. (c) Time-dependent phosphorescence decay of Host-CS/G-TPA, Host-CS/G-DMAC and Host-CS/G-PTZ at 645-655 nm. (d) Time-dependent phosphorescence decay of Host-CS/G-TPA, Host-CS/G-DMAC and Host-CS/G-PTZ at 730-750 nm.

**NMR spectra**

**Figure S29**. ^1^H NMR spectrum of Host-TPA.

**Figure S30**. ^13^C NMR spectrum of Host-TPA.

**Figure S31**. ^1^H NMR spectrum of Host-DMAC.

**Figure S32**. ^13^C NMR spectrum of Host-DMAC.

**Figure S33**. ^1^H NMR spectrum of Host-PTZ.

**Figure S34**. ^13^C NMR spectrum of Host-PTZ.

**Figure S35**. ^1^H NMR spectrum of Host-CS.

**Figure S36**. ^13^C NMR spectrum of Host-CS.

**Figure S37**. ^1^H NMR spectrum of BT.

**Figure S38**. ^13^C NMR spectrum of BT.

**Figure S39**. ^1^H NMR spectrum of 2Br-BT.

**Figure S40**. ^13^C NMR spectrum of 2Br-BT.

**Figure S41**. ^1^H NMR spectrum of G-TPA-CH_3_.

**Figure S42**. ^13^C NMR spectrum of G-TPA-CH_3_.

**Figure S43**. ^1^H NMR spectrum of G-DMAC-CH_3_.

**Figure S44**. ^13^C NMR spectrum of G-DMAC-CH_3_.

**Figure S45**. ^1^H NMR spectrum of G-PTZ-CH_3_.

**Figure S46**. ^13^C NMR spectrum of G-PTZ-CH_3_.

**Figure S47**. ^1^H NMR spectrum of G-CS-CH_3_.

**Figure S48**. ^13^C NMR spectrum of G-CS-CH_3_.

**Figure S49**. ^1^H NMR spectrum of G-TPA.

**Figure S50**. ^13^C NMR spectrum of G-TPA.

**Figure S51**. ^1^H NMR spectrum of G-DMAC.

**Figure S52**. ^13^C NMR spectrum of G-DMAC.

**Figure S53**. ^1^H NMR spectrum of G-PTZ.

**Figure S54**. ^13^C NMR spectrum of G-PTZ.

**Figure S55**. ^1^H NMR spectrum of G-CS.

**Figure S56**. ^13^C NMR spectrum of G-CS.
